# An efficient neural network of cooperating serotonergic and noradrenergic neurons in modulating sudden unexpected death in epilepsy

**DOI:** 10.7150/ijbs.114659

**Published:** 2025-10-10

**Authors:** Qing Xu, XiaoXia Xu, LeYuan Gu, YaXuan Wu, Yue Yang, ZhuoYue Zhang, ZiWen Zhang, XuanYi Di, XiTing Lian, Qian Yu, YuLing Wang, HaiXiang Ma, WeiHui Shao, Lu Liu, JiaXuan Gu, Fei Tong, HongHai Zhang

**Affiliations:** 1 Department of Anesthesiology, Zhejiang University School of Medicine, Hangzhou, 310006, China.; 2 Department of Anesthesiology, Affiliated Hangzhou First People's Hospital, Westlake University School of Medicine, Hangzhou, 310006, China.; 3 Department of Anesthesiology, the Fourth Clinical School of Medicine, Zhejiang Chinese Medical University, Hangzhou, 310006, China.

**Keywords:** sudden unexpected death in epilepsy, seizure-induced respiratory arrest, dorsal raphe nucleus, locus coeruleus, venlafaxine

## Abstract

Sudden unexpected death in epilepsy (SUDEP) is a critical concern, with seizure-induced respiratory arrest (S-IRA) being a major contributing factor. The serotonergic (5-HT) and noradrenergic (NE) neurons have emerged as key modulators of SUDEP, yet the network-level interactions and specific mechanisms underlying their protective roles remain poorly defined. This study is the first to demonstrate a synergistic effect of 5-HT and NE in mitigating S-IRA and SUDEP using DBA/1 mice. Through a combination of pharmacological interventions, calcium signal recordings, and optogenetics, results show that elevating 5-HT and NE levels via 5-hydroxytryptophan and the norepinephrine reuptake inhibitor atomoxetine significantly reduced SUDEP incidence, with evidence of a robust synergistic interaction. Furthermore, venlafaxine, a selective serotonin-norepinephrine reuptake inhibitor, enhances the cooperative regulation of 5-HT and NE, further supporting their combined protective role. Crucially, the dorsal raphe-locus coeruleus-pre-Bötzinger complex (DR-LC-PBC) neural network is demonstrated as a critical pathway underlying this modulation. Targeted administration of the 5-HT2A/NE α-1 receptor antagonist and agonist into the PBC reveal their pivotal roles in mediating the protective effects of 5-HT and NE. Our study reveals that serotonergic and noradrenergic systems synergistically regulate SUDEP, and further identifies that the DR-LC-PBC neural circuit exerts a protective effect through activation of 5-HT2A and NE-α1 receptors within the PBC.

## Introduction

Epilepsy ranks as the second most prevalent neurological disorder, trailing only behind stroke, with a global prevalence of approximately 70 million individuals 1,2. Recent investigations have highlighted that the risk of Sudden Unexpected Death in Epilepsy (SUDEP) in pediatric patients is approaching that of adults, while the likelihood escalates during pregnancy among epileptics 3. Furthermore, SUDEP emerges as the primary contributor to mortality among young adults with epilepsy, exhibiting an incidence rate two to three times higher than other conditions leading to sudden death in this demographic 4. SUDEP represents a highly critical yet frequently overlooked complication among this patient population, constituting the foremost direct cause of premature mortality associated with epilepsy 5. The precise mechanisms underlying SUDEP remain elusive, though they are postulated to involve intricate interactions among autonomic nervous system dysfunction, alterations in brainstem function, cardiorespiratory failure, and widespread suppression of postictal electroencephalographic activity. Among the identified risk factors, generalized tonic-chronic seizures (GTCS) and nocturnal seizures stand out as pivotal 6,7. Specifically, the neuronal networks activated during GTCS may impair brainstem respiratory or autonomic control centers, triggering hypoventilation, apnea, and cardiovascular collapse, ultimately culminating in patient demise. Recent scholarly endeavors have increasingly emphasized the significance of apnea and cardiac dysfunction as potential pivotal mechanisms in SUDEP. In particular, seizure-induced respiratory arrest (S-IRA) has emerged as a primary precipitating factor in numerous fatal cases 7-9. Consequently, there is an urgent need within the research community to elucidate the intricate mechanisms of SUDEP and devise strategies to mitigate or avert its occurrence, thereby offering tangible clinical translational benefits.

5-Hydroxytryptamine (Serotonin, 5-HT) plays a pivotal role in the central nervous system (CNS), encompassing the regulation of appetite, sleep, memory, learning, emotion, and behavior 10,11,12. Notably, 5-HT emerges as a potential key player in preventing the onset and progression of SUDEP 13. Studies suggest that elevating 5-HT levels in the synaptic cleft with 5-HT reuptake inhibitors can mitigate S-IRA triggered by audiogenic seizures in DBA/1 mice 14. The Dorsal Raphe Nucleus (DR), the primary source of forebrain 5-HT innervation, harbors 5-HT neurons whose activity is intimately linked to epilepsy 15,16. In the preliminary stages of our research, the group has made a noteworthy observation in a DBA/1 mouse model of SUDEP, optogenetic stimulation of DR 5-HT neurons has been found to markedly diminish the occurrence of S-IRA, indicating a promising therapeutic avenue 17. Norepinephrine (NE), another crucial neurotransmitter, is synthesized and secreted primarily by postganglionic sympathetic and noradrenergic neurons within the brain, with the latter responsible for its release. The Locus Coeruleus (LC), a major NE-synthesizing and releasing nucleus in the brain, influences respiratory and circulatory control via projections to the Nucleus Tractus Solitarius (NTS) 18-20. Our previous findings indicated that enhancing peripheral and central NE levels markedly decreases SUDEP incidence in DBA/1 mice, and with LC-NE neurons being of paramount importance, and deficient synthesis of NE and norepinephrinergic neurotransmission contributed to S-IRA, underscoring norepinephrine α-1 receptor (NEα-1R) as a potential therapeutic target for the prevention of SUDEP 21,22. Both 5-HT and NE, as major neurotransmitters within the brain and vital hormones in peripheral circulation, participate in regulating respiration, cardiac activity, sleep-wake cycles, and the autonomic nervous system, encompassing both sympathetic and parasympathetic components 23. These neurotransmitters are intimately associated with SUDEP. However, whether 5-HT and NE are intrinsically connected when they play the same role, and whether there are common pathways and bridges in SUDEP, still unknown.

**Figure 1:**
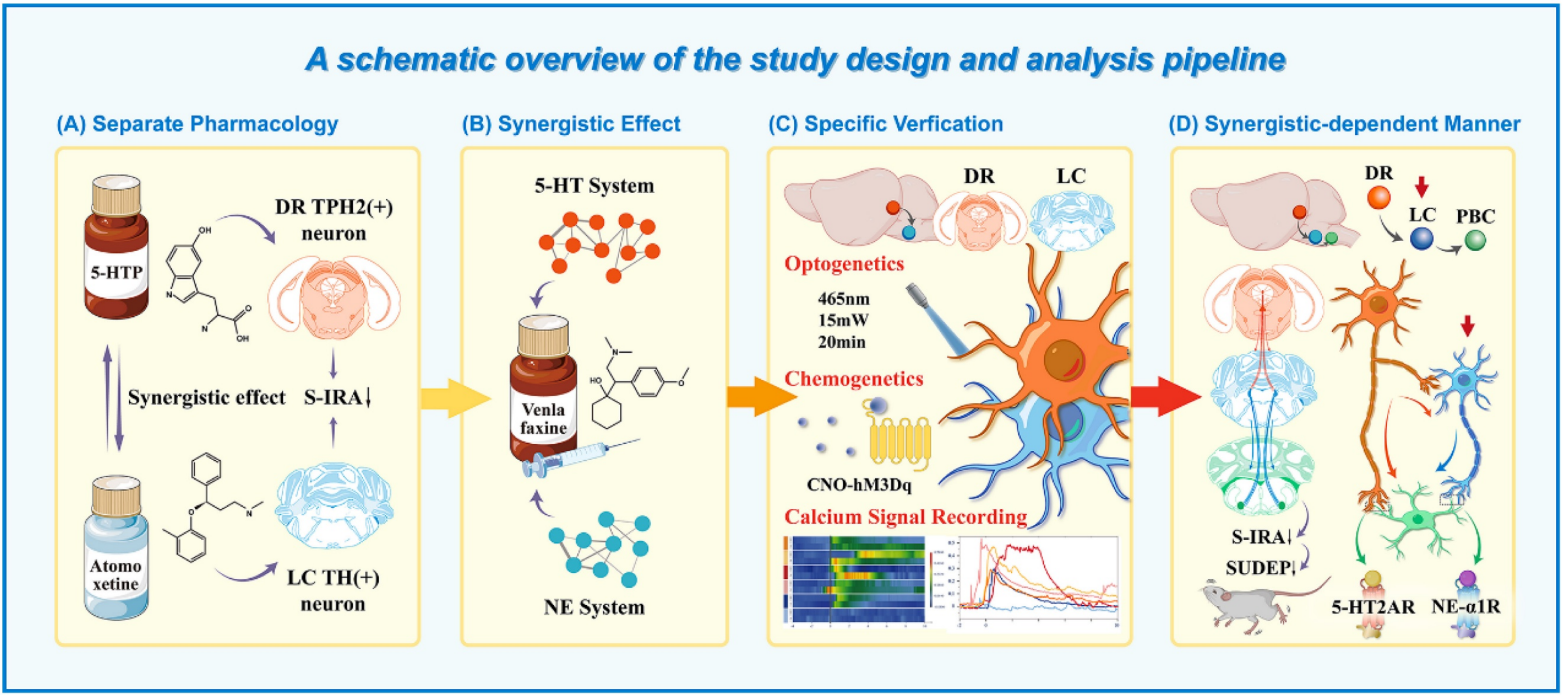
** A schematic overview of the study design and analysis pipeline. (A)** Schematic diagram of 5-HTP targeting TPH2 neurons in DR to increase the levels of 5-hydroxytryptophan and Atomoxetine targeting TH neurons in LC to increase NE and observing their effects on S-IRA. **(B)** Schematic diagram of venlafaxine targeting TPH2 neurons in DR and TH neurons in LC to simultaneously increase the levels of 5-hydroxytryptophan and NE and observing the effects on S-IRA. **(C)** Schematic diagram of the application of calcium signaling fiber photometry, optogenetic modulation and Chemogenetic modulation for specific verification. **(D)** Schematic diagram of activation of DR and LC through 5-HT2A and NE-α1 receptors in PBC in a synergistic-dependent manner to attenuate SUDEP.

Given the vital role of monoamine inhibitors in modulating SUDEP, we hypothesized that the central noradrenergic and serotonergic circuits may effectively co-regulate SUDEP in a certain manner. To verify the hypothesis, we designed the experiments as depicted in Figure 1 and employed acoustic stimulation and PTZ models to explore the mechanisms and interactions between 5-HT and noradrenergic neurons in modulating SUDEP 24,25. DBA/1 mice were intraperitoneally injected with 5-hydroxytryptophan (5-HTP) and the NE reuptake inhibitor atomoxetine to increase the levels of 5-HTP and NE. It was found that both 5-HTP and Atomoxetine could significantly reduce the incidence of S-IRA in mice, and they had a synergistic effect. Then we chose venlafaxine, a Selective Serotonin-Norepinephrine Reuptake Inhibitor (SNRIs) and found that it can also significantly reduce the incidence of S-IRA, which is consistent with another research 10. This finding implies a complex interplay between these neurotransmitters that could be crucial for understanding the mechanisms underlying SUDEP and developing targeted therapeutic strategies.

Given that the DR and the LC are the primary sites for the synthesis of 5-HT and NE in the brain, both play a significant role in the occurrence of SUDEP, based on our previous findings. The activity of DR^5-HT^ and LC^NE^ neurons were recorded by calcium signaling recording, and regulated by optogenetics and chemogenetics. Then, we investigated whether DR and LC regulate SUDEP in a Synergistic-Dependent manner by applying Para-chlorophenylalanine (PCPA) to reduce 5-HT levels and N-(2-chloroethyl)-N-ethyl-2-bromobenzylamine hydrochloride (DSP-4) to degrade reduce LC^NE^ neurons. We previously observed that the pre-Bötzinger complex (PBC), known for its role in regulating respiratory rhythms, was involved in the acoustic stimulation of DBA/1 mice or the S-IRA induced by pentadiazole (PTZ) injection, and found that 5-HT2AR, located in PBC, plays an important role in SUDEP 24. Therefore, we chose the 5-HT2AR/NE α-1R antagonist and agonist microinjected into PBC, and found that the combination of subtherapeutic doses of KET and prazosin, the 5-HT2AR/NE α-1R antagonist, reversed the S-IRA-reducing effects of venlafaxine.

Our findings underscored that both the 5-HT and NE neuronal systems are involved in the development of SUDEP, with a synergistic effect observed. We also found that the intricate interplay between DR-LC-PBC, and their critical roles in regulating SUDEP. It also suggested the clinical translational potential of venlafaxine, a SNRIs antidepressant drug, in treating patients with comorbid epilepsy and depression, thereby preventing SUDEP.

## Materials and Methods

### Animals

All experimental procedures adhered to the Guidelines for the Care and Use of Laboratory Animals issued by the National Institutes of Health and were approved by the Animal Advisory Committee of Zhejiang University. DBA/1 mice, aged 7-8 weeks and weighing 15-20g, were purchased from Vital River Laboratories, SLAC Laboratory Animal, and Jihui Laboratory Animal. All mice were housed in a specific pathogen-free (SPF) barrier facility at the Animal Center of the School of Medicine, Zhejiang University. Mice had *ad libitum* access to water and food, and were maintained under optimal temperature and humidity conditions. For the acoustic stimulation model, the housing environment was kept quiet to avoid excessive noise levels. Prior studies have shown that gender does not affect the incidence rate in DBA/1 mice 26. Also, we selected DBA/1 mice because they exhibit a consistently high incidence of S-IRA and SUDEP, making them a robust and sensitive model for mechanistic and interventional studies. In contrast, C57BL/6 mice show a much lower susceptibility to S-IRA, which limits statistical power and reduces sensitivity for detecting treatment effects 26 (Fig. 13 K-L). Hence, both male and female DBA/1 mice were selected for this study based on breeding availability.

The generation of TH-Cre transgenic mice with a DBA/1 genetic background was accomplished through a series of crosses and backcrosses involving TH-Cre transgenic mice originally on a C57BL/6J background and DBA/1 mice. The initial C57BL/6J TH-Cre transgenic mice, generously provided by the Jackson Laboratory (strain B6. Cg-7630403G23RikTg(TH-Cre)1Tmd/J), were hemizygous for the 7630403G23RikTg (TH-Cre)1Tmd allele and served as the foundational Cre animal model. Utilizing marker-assisted accelerated backcrossing, hemizygous transgenic DBA/1 TH-Cre mice (hereafter referred to as transgenic DBA/1 mice) were derived from crosses between these hemizygous transgenic C57BL/6J TH-Cre mice and wild-type DBA/1 mice. Congenic transgenic DBA/1 mice, possessing an approximately 100% DBA/1 genetic background, were subsequently produced from the progeny of the fifth backcross generation. These hemizygous transgenic DBA/1 mice were further crossed with wild-type DBA/1 mice to yield the experimental cohort of hemizygous transgenic DBA/1 mice utilized in the subsequent studies.

### PCR-based genotyping for TH-Cre transgenic mice

A 3-5 mm segment was excised from the toe or tail of each transgenic mouse 1 week after birth. The tissue was placed into a sterile EP tube and centrifuged to pellet it at the bottom. A solution was prepared by mixing AD1 Buffer and AD2 Buffer in a 4:1 ratio (e.g., 10 µl of AD1 Buffer and 2.5 µl of AD2 Buffer). This mixture was added to the centrifuge tube containing the tissue, followed by a 10-minute incubation at room temperature. The mixture was then heated at 95 °C for 3 min. Subsequently, AD3 Buffer was added, and the solution was mixed thoroughly. The sample was stored at 4 ºC and used for PCR within one week. For PCR, the reaction mixture was prepared as follows: 4µl of primers, 12.5 µl of Taq enzyme, 7 µl of ddH2O, and 1.5 µl of the sample. The prepared samples were placed into a PCR instrument for reaction. In preparation for gel electrophoresis, 3 g of agarose were combined with 100 ml of TAE buffer in a conical flask. The mixture was heated in a microwave oven for 2 min until it boiled, then removed and mixed thoroughly. When the temperature reached 60 °C, 10 µl of dye was added, and the solution was poured into a gel mold. Bubbles were removed, and an electrophoresis loading comb was inserted. After the gel had solidified, the comb was removed and samples were loaded into the wells. The electrophoresis apparatus was set to a constant voltage of 170 V for 18 min. Following electrophoresis, the gel was developed, and bands were analyzed to identify DBA/1 background TH-Cre mice.

### Seizure induction and resuscitation

The mouse model of S-IRA induced by acoustic stimulation was established as we previously described 17,18. To establish a model of audiogenic seizures and susceptibility to S-IRA, DBA/1 mice were subjected to continuous acoustic stimulation for 3-5 days between postnatal days 26-28. The mice were placed in a cylindrical plexiglass container within an acousticproof chamber and exposed to acoustic stimulation from an electric bell (96 dB SPL, Zhejiang People's Electronics, China). The stimulation continued until the onset of audiogenic seizures (AGSz) or until S-IRA was observed, with a maximum stimulation duration of 60 seconds. Upon the occurrence of S-IRA, resuscitation was promptly initiated. The rodent ventilator (YuYan Instruments, Shanghai, China) was used to restore normal breathing within 5 seconds of the last respiratory gasp. The ventilator was set to deliver 180 breaths per minute, with an inspiratory-to-expiratory (I/E) ratio of 1:1. 5, and a tidal volume of 1 cc of air. For PTZ-induced SUDEP model, DBA/1 mice were administered a single intraperitoneal injection of PTZ (Cat # P6500; Sigma-Aldrich, St. Louis, MO, USA) at a dose of 75 mg/kg to induce generalized seizures (GSz) and S-IRA. Mice exhibiting S-IRA were resuscitated within 5 seconds of the last respiratory gasp using a rodent respirator 27.

### Pharmacological intervention experiments

All experiments were conducted in a controlled animal laboratory, maintaining appropriate temperature, humidity, adequate lighting, and minimal noise. The mice, primarily littermates, had an age difference of no more than one week. To minimize stress, cages were left unchanged for 3-4 days before experiments, and the mice were gently stroked daily to familiarize them with the experimenter's scent and handling. This acclimatization also facilitated the attachment of optical fibers to their heads for testing. On the day of behavioral testing, mice were placed in the testing room 30-60 min in advance to reduce nonspecific stress and other confounding factors. For acoustic stimulation model, the susceptibility of DBA/1 mice to AGSz and S-IRA was confirmed 24 h before the experiments as described previously 23. Mice behaviors were recorded using cameras in an acousticproof chamber. The videos were later analyzed to assess S-IRA incidence, AGSz latency, duration of wild running and clonic seizures (W+C), duration of tonic-clonic seizures, and seizure scores.

#### 5-HTP and atomoxetine reversed the S-IRA induced by acoustic stimulation or PTZ in DBA/1 mice

5-HTP (Cat #107751, Sigma-Aldrich, USA), an intermediate metabolite of the essential amino acid L-tryptophan (LT) in the serotonin biosynthetic pathway, enhances serotonin levels in both central and peripheral nervous systems. Clinically, it is often utilized as an antidepressant, appetite suppressant, and sleep aid 28. Atomoxetine (Cat #Y0001586, Sigma-Aldrich, USA), a selective norepinephrine (NE) reuptake inhibitor, targets the presynaptic NE transporter (NET), blocking NE reuptake and increasing its levels in the synaptic cleft. It is currently used in the treatment of attention deficit hyperactivity disorder (ADHD) in children, adolescents, and adults 29. Commonly used for treating ADHD in children, adolescents, and adults, both 5-HTP and atomoxetine were dissolved in 0.9% saline solution.

For acoustic stimulation model, 5-HTP was administered 1 h before acoustic stimulation, and DBA/1 mice were intraperitoneal (IP) injected with 5-HTP (100 mg/kg or 125 mg/kg, treatment group) or saline (control group, DB01-1-0701, China). Atomoxetine was administered 2 h before acoustic stimulation, and DBA/1 mice received IP injections of atomoxetine (5, 10, 15, or 20 mg/kg, treatment group) or saline (control group).

In the PTZ model, DBA/1 mice received IP injections of either 5-HTP (100 mg/kg or 125 mg/kg) or saline for pre-treatment on day 1. On day 2, the same pre-treatment protocol was followed, with an additional IP injection of 5-HTP (100 mg/kg or 125 mg/kg) or saline administered 1 h before the IP injection of PTZ (Cat #P6500, Sigma-Aldrich) at 75 mg/kg. Atomoxetine (5 mg/kg or 15 mg/kg) or saline was administered 2 h before the IP PTZ injection (75 mg/kg).

#### Effect of venlafaxine on acoustic stimulation or PTZ model-induced S-IRA in DBA/1 Mice

Venlafaxine (PHR1736, Sigma-Aldrich, USA), a 5-HT and NE reuptake inhibitor, blocks the reuptake of 5-HT and NE neurotransmitters. Clinically, used for treating major depressive disorder and generalized anxiety disorder, venlafaxine behaves like Selective Serotonin Reuptake Inhibitors (SSRIs) at low doses, affecting only 5-HT receptors exclusively. At medium to high doses, venlafaxine also targets NE receptors, while showing low affinity for histamine and cholinergic receptors 30. venlafaxine was dissolved in 0.9% saline.

In the acoustic stimulation model, the acoustic stimulation, DBA/1 mice were exposed to acoustic stimulation and then received IP injections of venlafaxine at doses of 5, 15, 25, 50, 75, or 100 mg/kg, or saline 30 min after the acoustic exposure. In the PTZ model, DBA/1 mice were intraperitoneally injected with different doses of venlafaxine (5, 15, 25, 50, 75, or 100 mg/kg) or saline, and then intraperitoneally injected with PTZ (75 mg/kg) 30 min later.

#### Effect of KET/Prazosin on acoustic stimulation or PTZ model-induced S-IRA in DBA/1 Mice

KET (Cat #8006, Sigma-Aldrich, USA) is a selective serotonin receptor antagonist that primarily targets the 5-HT2A receptor, inhibiting its activity. Prazosin (Cat #P7791, Sigma-Aldrich, USA) acts as an NE-α1R antagonist, blocking norepinephrine effects on α1-receptors. Prazosin is commonly used to manage hypertension, benign prostatic hyperplasia, and post-traumatic stress disorder 31. Both KET and prazosin were dissolved in 25% Dimethylsulfoxide (DMSO).

For the acoustic stimulation model, DBA/1 mice were administered venlafaxine (25 mg/kg) or saline via IP injection 30 min prior to acoustic stimulation. In the experimental group, mice received KET (20 mg/kg) 15 min before acoustic stimulation and prazosin (0.01 mg/kg) 30 min before acoustic stimulation. The control group received IP injections of 25% DMSO (SHBK2703, Sigma-Aldrich, USA) at both 15 min and 30 min before acoustic stimulation. In the PTZ model, DBA/1 mice were administered an IP injection of venlafaxine at 25 mg/kg or saline 30 min before the PTZ injection (75 mg/kg). Similarly, experimental mice received an IP injection of KET at a 20 mg/kg 15 min before the PTZ injection, and an IP injection of prazosin at 0.01 mg/kg 30 min before. The control group received IP injections of 25% DMSO at both 15 min and 30 min prior to the PTZ injection.

#### Effect of PCPA/DSP-4 on PTZ-induced venlafaxine-mediated S-IRA inhibition in DBA/1 Mice

PCPA (C3635, Sigma-Aldrich, USA) acts as a selective and irreversible inhibitor of TPH (Tryptophan Hydroxylase), the rate-limiting enzyme in 5-HT synthesis. By inhibiting tryptophan hydroxylation, PCPA significantly reduces 5-HT levels in both peripheral and central systems, and is commonly used in animal behavioral studies such as sleep-wake, analgesia, and epilepsy 32,33. DSP-4 (C8417, Sigma-Aldrich, USA) selectively targets the noradrenergic system in the LC. It readily crosses the blood-brain barrier and forms a reactive aziridine derivative, which accumulates in noradrenergic nerve terminals via the NE transporter. Within nerve terminals, aziridine derivative reacts with cellular components, resulting in the destruction of nerve terminals 21,34. Our previous studies have also demonstrated that DSP-4 significantly reduces noradrenergic neuron numbers in the LC 18. Both PCPA and DSP-4 were dissolved in 0.9% saline.

DBA/1 mice received with IP injections of either PCPA (800 mg/kg) or Saline, administered either as a single dose 1 day prior or daily for 5 consecutive days. On the final day, mice got an additional IP injection of either PCPA (800 mg/kg) or Saline 2.5 h before PTZ injection. Mice were also given an intraperitoneal injection of venlafaxine (25 mg/kg) or saline 30 min before PTZ injection. For DSP-4 treatment, mice were pretreated with a single IP injection of DSP-4 (50 mg/kg) or saline. Mice were injected intraperitoneally with venlafaxine (25 mg/kg) or saline 30 min before IP injection of PTZ (75 mg/kg).

#### Effect of TCB-2/Phenylephrine on PTZ model-induced S-IRA in DBA/1 Mice

TCB-2 ((4-Bromo-3,6-dimethoxybenzocyclobuten-1-yl) methylamine hydrobromide) is a high-affinity and selective 5-HT2A receptor agonist and acts as a potent agonist for the 5-HT2A and 5-HT2C receptors with exceptional selectivity for the 5-HT2A receptor. TCB-2 has been demonstrated to induce head twitches and hypothermia in mice following intraperitoneal administration 43.

Phenylephrine is a selective alpha-1 adrenergic receptor agonist, and mediates vasoconstriction and mydriasis depending on the route and location of administration, with systemic exposure leading to increased systolic and diastolic pressure as well as peripheral vascular resistance. Previous research has demonstrated phenylephrine's involvement in modulating seizure activity and respiratory function. Studies have shown that phenylephrine can potentiate anticonvulsant effects when combined with other antiepileptic drugs, specifically enhancing the anticonvulsant activity of diazepam while neutralizing its sedative effects 44.

In the PTZ model, DBA/1 mice received IP injections of TCB-2 (5, or 10 mg/kg) 30 minutes before PTZ injection (75 mg/kg), or phenylephrine hydrochloride (1, 3, or 5 mg/kg) 1 minute before PTZ injection. The behaviors were recorded and analyzed for S-IRA incidence, seizure latency, seizure duration, and seizure severity scores as described in our previous methods.

### Immunohistochemistry

Immunohistochemistry was performed as described previously 17. Mice were anesthetized with an intraperitoneal injection of 1% pentobarbital sodium (50 mg/kg), then perfused with phosphate-buffered saline (PBS) and 4% paraformaldehyde (PFA) in PBS. Following perfusion, brains were harvested and fixated in 4% PFA overnight. After fixation, the brains were saturated in 30% sucrose for 24 h until they sank to the bottom. Dehydrated tissue was cut into 35 or 40 μm thick sections with a freezing microtome. After washing with PBS, the brain sections were blocked by incubation for 2 h at room temperature in blocking solution containing 10% normal donkey serum (017-000-121, Jackson Immuno Research, West Grove, PA), 1% bovine serum albumin (A2153, Sigma-Aldrich), and 0.3% Triton X-100 in PBS. The primary antibodies used mouse anti-TH (1:1000; MAB318, Merck-Millipore), mouse anti- TPH2 (1:500; T0678, Sigma-Aldrich), and rabbit anti-c-Fos (1:1000, 2250S, Cell Signaling Technology), and the secondary antibodies used donkey anti- mouse Alexa 546 (1:1000; A10036, Thermo Fisher Scientific), donkey anti-mouse Alexa 488 (1:1000; A21202, Thermo Fisher Scientific), Goat anti-rabbit Cy5 (1:1000; A10523, Thermo Fisher Scientific). Finally, the brain slices were stained with DAPI for 7-10 min and mounted with anti-fluorescence attenuating tablet. Confocal images were acquired using the VS120 virtual slide system and Nikon A1R Confocal Microscope. The images were then analyzed using ImageJ and NIS-Elements Viewer.

### Stereotaxic surgery and viral Injection

DBA/1 mice were anesthetized with 1% pentobarbital sodium (50 mg/kg) and head-fixed in a stereotaxic apparatus (68,018, RWD Life Science, Shenzhen, China), as previously described 17. The body temperature of the anesthetized mouse was maintained at a constant 37 °C using a heating pad throughout the surgery. The hair on the mouse's head was shaved, and the mouse was secured in a stereotaxic apparatus using ear bars and a nose clamp, ensuring that the respiratory tract remained unobstructed. To prevent irreversible damage to the mouse's vision from bright light, erythromycin eye ointment was applied to the surface of the mouse's eyeballs, followed by a covering of sterile cotton balls for protection. After disinfecting with alcohol, the mouse head skin was cut, and the connective tissue on the surface of the skull was wiped with an alcohol-soaked cotton ball. For microinjection of drugs, cannulas (RWD Life Science) were implanted for intracerebroventricular (ICV) or PBC injection. Virus was microinjected via a gauge needle for the specification of 10 ul (cat# 60700010, Gao Ge, ShangHai, China) by an ultra-micropump (160494 F10E, WPI) at a rate of 40 nL/min using the stereotaxic coordinates.

Anterograde tracing virus HSV-1 strain H129 (Herpes simplex virus-1 H129, HSV-1 H129) possesses the characteristic of anterograde trans-synaptic transport across multiple synapses, enabling it to label entire neural networks. Following viral infection, it can cross one synapse within 24-36 h, two synapses within 36-48 h, and up to three synapses within 60-72 h. It is important to note that HSV-1 H129 virus injection is toxic to mice, with a typical survival time of 3-5 days after injection. In this experiment, the HSV-1 H129 infection time was set at 36-48 hours. In contrast, the retrograde tracer virus CTB-555 can be taken up from axonal terminals and retrogradely transported to label neuronal cell bodies, with an infection time of 1-2 weeks.

### Optogenetics and calcium signaling fiber optic recording

DBA/1 background TH-Cre transgenic mice were employed for optogenetics, as previously described 23. The pAAV-TPH2 PRO-ChETA-EYFP-WPRES-PAS (100 nL, Shengbo Biomedical Technology, Shanghai, China) was microinjected into the DR (AP -4.55 mm, ML -0.44 mm, DV -2.80 mm, 10° right) and pAAV-CAG-DIO-CHETA-EGFP (100 nL, Shengbo Biomedical Technology, Shanghai, China) or mTH-Cre-AAV plus AAV-EF1a-DIO-hChR2 (H134R)-eYFP (100 nL, Brain VTA Technology, Wuhan, China) was microinjected into the LC (AP -5.45 mm; ML ± 0.9 mm; DV-3.65 mm). Then, a 200-mm optic fiber optical fiber (FOC-W-1.25-200-0.37-3.0, Inper, Hangzhou, China) was implanted above the area. Three weeks after the injection of the virus, the photostimulation (blue-light, 465 nm, 20 Hz, 20-ms pulse width, 15 mW, and 20 min) was delivered by the laser (B12124, Inper) fiber, which was proved to be effective on the suppression of S-IRA 17. And mice received PTZ at 15 min during the light stimulation period. Behavioral observations and recordings were conducted, and immunohistochemical analysis was performed to quantify neuronal activation within the two nuclei.

For calcium signal recording of the DR, AAV2/9-mCaMKIIa-GCaMP6f-WPRE-pA (100 nL, Brain VTA Technology) was microinjected into the DR. In order to record calcium signal in the LC and PBC, rAAV-DBH-GCaMP6m-WPRE-hGH pA (100 nL, Brain VTA Technology) was microinjected into the LC or PBC (AP -6.80 mm, ML ± 1.25 mm, DV 4.95 mm). Then, the optical fiber was implanted above the area. Three weeks after the injection of the virus, the calcium signal was recorded by fiber photometry system (C11946, Inper) with a 488-nm diode laser. The recording was started 20 min before IP injection of PTZ (75 mg/kg) and stopped 60 min after PTZ injection. In this study, various concentrations of venlafaxine (1.25 mg/ml, 2.5 mg/ml, 6.25 mg/ml, 12.5 mg/ml, and 25 mg/ml, 2.0 μl) or Saline (2.0 μl) were injected ICV through a cannula, while calcium signals were recorded. The time points were aligned with the onset of seizures, and the fluorescence intensity changes in DR^5-HT^ and LC^NE^ neurons were expressed as ΔF/F [ (F-F0)/F0, F represents the current fluorescence intensity and F0 represents the baseline fluorescence intensity].

### Chemogenetics

Chemogenetics was performed as described previously 35. Following bilateral LC injection with a 100 nL volume of mixture of mTH-Cre-AAV/DBH-Cre and pAAV-EF1a-DIO-hM3D-mCherry viruses (1:3, 5.00 × 10¹² vg/mL, Brain VTA Technology) the mice were allowed to recover for a minimum of 3 weeks before treatment with Clozapine-N-oxide (CNO; HY-17366, MedChemExpress). CNO was dissolved in saline and administered to mice via IP injection. Two doses of CNO (0.5 mg/kg and 1 mg/kg) were evaluated, ultimately determining 1 mg/kg as the optimal dose for subsequent intraperitoneal administrations. After chemogenetic manipulation, immunohistochemical analysis was employed to measure the expression levels of TH-hM3Dq in the LC.

### Quantification and statistical analysis

Experimental data were presented as mean ± standard error of the mean (SEM). Prior to data analysis, data tested for normality using the Shapiro-Wilk test and for homogeneity of variance with Levene's test. For comparing two groups, if the data follow a normal distribution, a Student's t-test is applied, including the independent samples t-test, which assumes both variance homogeneity and normal distribution. If the data do not meet these criteria, the Mann-Whitney U test or the Wilcoxon signed-rank test is utilized. When comparing three or more groups, a One-way ANOVA is conducted if the data are normally distributed and variances are homogeneous. A P-value < 0.05 is considered statistically significant. Statistical analyses were performed using GraphPad Prism™ 8.0 and SPSS version 23.0.

## Results

### Co-enhancement of 5-HT and NE neurotransmission in the brain significantly reduced the incidence of S-IRA evoked by acoustic stimulation or PTZ injection, exhibiting a synergistic effect

**Figure 2:**
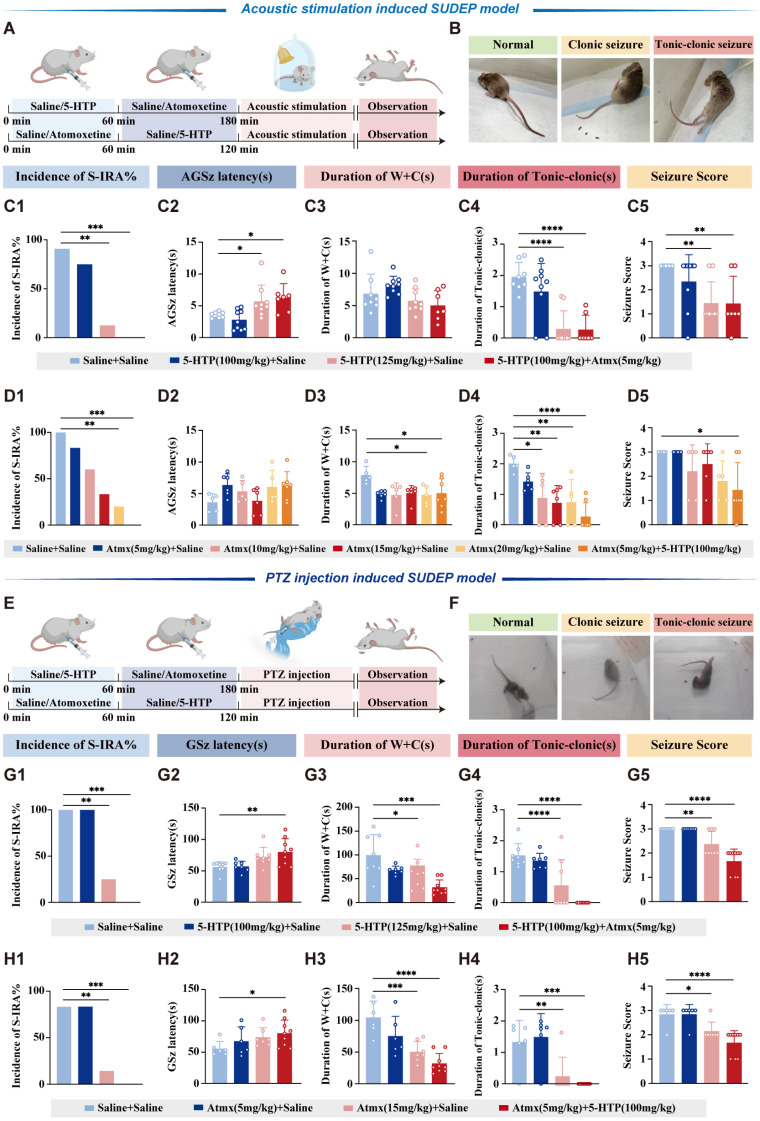
** Elevated levels of 5-HT and NE demonstrate synergistic effects in reducing the incidence of S-IRA induced by acoustic stimulation and PTZ injection.** Schematic illustration of the observations in the DBA/1 mice induced by acoustic stimulation following IP injection of 5-HTP or Atomoxetine with or without Atomoxetine. Representative images of mice in the stage of normal, clonic seizure, and tonic-clonic seizure caused by acoustic stimulation. **(C1-5)** The incidence of S-IRA evoked by acoustic stimulation, the duration of tonic-clonic seizures and the seizure score in the group with 125mg/kg 5-HTP and the group with 100mg/kg 5-HTP + 5mg/kg Atomoxetine were significantly reduced compared to the group with Saline. The AGSz latency in the group with 125mg/kg 5-HTP and the group with 100mg/kg 5-HTP + 5mg/kg Atomoxetine was markedly higher than the group with Saline. No obvious differences were observed between treatment groups in the duration of wild running and clonic seizures (*p* > 0.05). **(D1-5)** The incidence of S-IRA evoked by acoustic stimulation and the duration of wild running and clonic seizures in the group with 20mg/kg Atomoxetine and the group with 5mg/kg Atomoxetine +100mg/kg 5-HTP were significantly reduced compared to the group with Saline. The duration of tonic-clonic seizures in the group with 10mg/kg Atomoxetine, 15mg/kg Atomoxetine, 20mg/kg Atomoxetine and the group with 5mg/kg Atomoxetine +100mg/kg 5-HTP was significantly reduced compared to the group with Saline. The seizure score in the group with 5mg/kg Atomoxetine +100mg/kg 5-HTP was markedly lower than the group with Saline. No obvious differences were observed between treatment groups in the AGSz latency (*p* > 0.05). **(E)** Schematic illustration of the observations in the DBA/1 mice induced by PTZ injection following IP injection of 5-HTP or Atomoxetine with or without Atomoxetine. **(F)** Representative images of mice in the stage of normal, clonic seizure, and tonic-clonic seizure caused by PTZ injection. **(G1-5)** The incidence of S-IRA evoked by PTZ, the duration of wild running and clonic seizures, the duration of tonic-clonic seizures and the seizure score in the group with 125mg/kg 5-HTP and the group with 100mg/kg 5-HTP + 5mg/kg Atomoxetine were significantly reduced compared to the group with Saline. The GSz latency in the group with 100mg/kg 5-HTP + 5mg/kg Atomoxetine was markedly higher than the group with Saline. **(H1-5)** The incidence of S-IRA evoked by PTZ, the duration of wild running and clonic seizures, the duration of tonic-clonic seizures and the seizure score in the group with 15mg/kg Atomoxetine and the group with 5mg/kg Atomoxetine + 100mg/kg 5-HTP were significantly reduced compared to the group with Saline. The GSz latency in the group with 5mg/kg Atomoxetine + 100mg/kg 5-HTP was markedly higher than the group with Saline. ****P < 0.0001; ***P < 0.001; **P < 0.01; *P < 0.05; Data are mean±SEM; i.p.: intraperitoneal injection

To verify the hypotheses of the interactive effects between 5-HT and NE proposed in the Fig. 1, we started with pharmacology experiments. DBA/1 mice were administered different doses of 5-HTP and atomoxetine via IP injection in acoustic stimulation or PTZ models (Fig. 2A-B, E-F). In both models, the incidence of S-IRA was significantly reduced when treated with high-dose 125mg/kg 5-HTP or 15-20 mg/kg atomoxetine alone. In contrast, the application of lower doses of 5-HTP or atomoxetine did not suppress the incidence of S-IRA. Intriguingly, combining ineffective doses of 100mg/kg 5-HTP and 5 mg/kg atomoxetine significantly reduced the incidence of S-IRA, prolonged the latency of AGSz seizures, decreased the duration of wild running and clonic seizures, the duration of tonic-clonic seizures and seizure scores, surpassing the effects observed with either drug alone (Fig. 2C-D, 2G-H). These results underscored the potential synergistic effect of concurrently enhancing 5-HT and NE neurotransmission.

### Venlafaxine reduced the S-IRA occurrence of SUDEP via synergistical activation of DR^5-HT^-LC^NE^ neural pathway

To further verify the relationship of the 5-HT and NE, venlafaxine was utilized to investigate the connection of DR and LC. Leveraging numerous studies and prior experiments, the DR and LC were identified as the key synthesis sites of 5-HT and NE, respectively, in the brain. Thus, DR and LC were selected as the target nuclei for hypothesis testing. Utilizing venlafaxine—a selective serotonin and norepinephrine reuptake inhibitor (SNRI)—to inhibit the reuptake of 5-HT and NE, this approach enhanced the availability of these neurotransmitters in neuronal synapses, optimizing their levels to explore their role in mitigating S-IRA.

**Figure 3:**
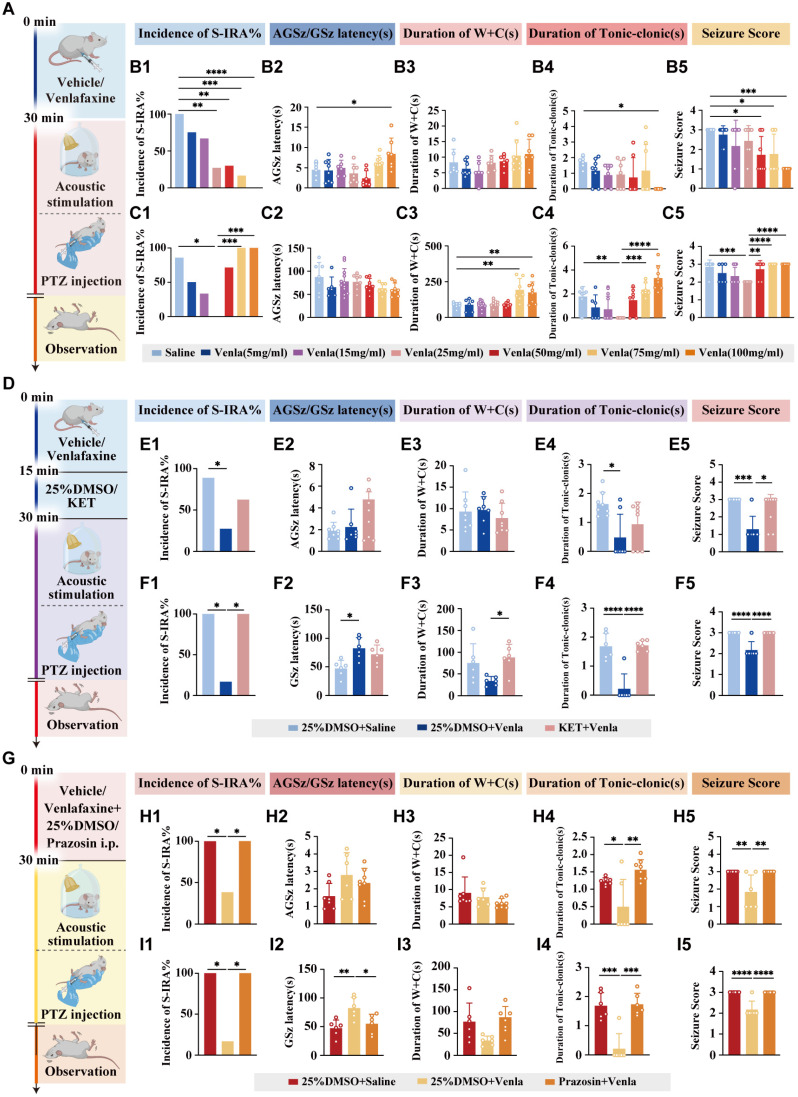
** venlafaxine significantly reduced the incidence of S-IRA induced by acoustic stimulation and PTZ injection, and 5-HT2AR and NE-α1R antagonists can reverse the effects. (A)** Schematic illustration of the observations in the DBA/1 mice induced by acoustic stimulation or PTZ injection following IP injection of different doses of venlafaxine. **(B1-5)** The incidence of S-IRA evoked by acoustic stimulation and the seizure score in the group with 25mg/kg venlafaxine, 50mg/kg venlafaxine, 75mg/kg venlafaxine and 100mg/kg venlafaxine were significantly reduced compared to the group with Saline. The AGSz latency in the group with 100mg/kg venlafaxine was markedly higher than the group with Saline. The duration of tonic-clonic seizures in the group with 100mg/kg venlafaxine was markedly higher than the group with Saline. The seizure score in the group with 50mg/kg venlafaxine, 75mg/kg venlafaxine and 100mg/kg venlafaxine was significantly decreased compared to the group with Saline. No obvious differences were observed between treatment groups in the duration of wild running and clonic seizures (*p* > 0.05). **(C1-5)** The incidence of S-IRA evoked by PTZ and the duration of tonic-clonic seizures in the group with 25mg/kg venlafaxine were markedly reduced compared to the group with Saline and the group with 75mg/kg venlafaxine and 100mg/kg venlafaxine. The duration of wild running and clonic seizures in the group with 75mg/kg venlafaxine and 100mg/kg venlafaxine was markedly higher than the group with Saline. The seizure score in the group with 25mg/kg venlafaxine was significantly decreased compared to the group with Saline and the group with Saline, 50mg/kg venlafaxine, 75mg/kg venlafaxine and 100mg/kg venlafaxine. No obvious differences were observed between treatment groups in the GSz latency (*p* > 0.05). **(D)** Schematic illustration of the observations in the DBA/1 mice induced by acoustic stimulation or PTZ injection following IP injection of venlafaxine and 5-HT2AR antagonist KET. **(E1-5)** The incidence of S-IRA evoked by acoustic stimulation, the duration of tonic-clonic seizures and the seizure score in the group with 25%DMSO + venlafaxine were markedly lower compared with other groups. No obvious differences were observed between treatment groups in the AGSz latency and the duration of wild running and clonic seizures (*p* > 0.05). **(F1-5)** The incidence of S-IRA evoked by PTZ injection, the duration of tonic-clonic seizures and the seizure score in the group with 25%DMSO + venlafaxine were markedly lower compared with other groups. The GSz latency in the group with 25%DMSO + venlafaxine was significantly increased compared to the group with 25%DMSO + Saline. The duration of wild running and clonic seizures in the group with 25%DMSO + venlafaxine was markedly decreased compared to the group with KET + venlafaxine. **(G)** Schematic illustration of the observations in the DBA/1 mice induced by acoustic stimulation or PTZ injection following IP injection of venlafaxine and NE-α1R antagonist prazosin. **(H1-5)** The incidence of S-IRA evoked by acoustic stimulation, the duration of tonic-clonic seizures and the seizure score in the group with 25%DMSO + venlafaxine were lower compared with other groups. No obvious differences were observed between treatment groups in the AGSz latency and the duration of wild running and clonic seizures (*p* > 0.05). **(I1-5)** The incidence of S-IRA evoked by PTZ injection, the duration of tonic-clonic seizures and the seizure score in the group with 25%DMSO + venlafaxine were markedly lower compared with other groups. The GSz latency in the group with 25%DMSO + venlafaxine was markedly increased compared with other groups. No obvious differences were observed between treatment groups in the duration of wild running and clonic seizures (*p* > 0.05). ****P < 0.0001; ***P < 0.001; **P < 0.01; *P < 0.05; Data are mean±SEM; i.p.: intraperitoneal injection

Thus, DBA/1 mice were intraperitoneally injected with different doses of venlafaxine or saline and then received acoustic stimulation 30 min later (Fig. 3A). Results showed a significant, dose-dependent reduction in S-IRA incidence compared to controls (P < 0.0001, Fig. 3 B1). To further mitigate the potential differences generated by the S-IRA induction method alone, a PTZ-induced epilepsy model was used revealing a significant reduction in S-IRA incidence at a venlafaxine dose of 25 mg/kg (P < 0.05, Fig. 3 C1). While there was no significant difference between the control group and other venlafaxine groups (P > 0.05, Fig. 3 C1). Unlike the acoustic stimulation model, the PTZ model exhibited a decrease in S-IRA incidence, tonic-clonic seizure duration, and seizure scores that initially decreased with increasing venlafaxine doses but later rose with the increase of venlafaxine dose, suggesting SUDEP induction differences between models.

Given previous studies showing that 5-HT2A and NE-α1 receptors are closely related to respiratory regulation, receptor involvement was investigated by administering the 5-HT2AR antagonist KET and the NE-α1R antagonist prazosin in both acoustic stimulation and PTZ injection models (Fig. 3 D, G). Results indicated that administration of KET subsequent to venlafaxine's IP injection, in contrast to the 25% DMSO + venlafaxine 25 mg/kg group, not only negated venlafaxine's efficacy in decreasing the prevalence of S-IRA but also reinstated the higher epilepsy scores (Fig. 3 E1-5, F1-5). Similarly, comparing with the same control group, prazosin reversed venlafaxine's suppressive impact on S-IRA, extending seizure duration and raising seizure severity scores (Fig. 3 H1-5, I1-5).

**Figure 4:**
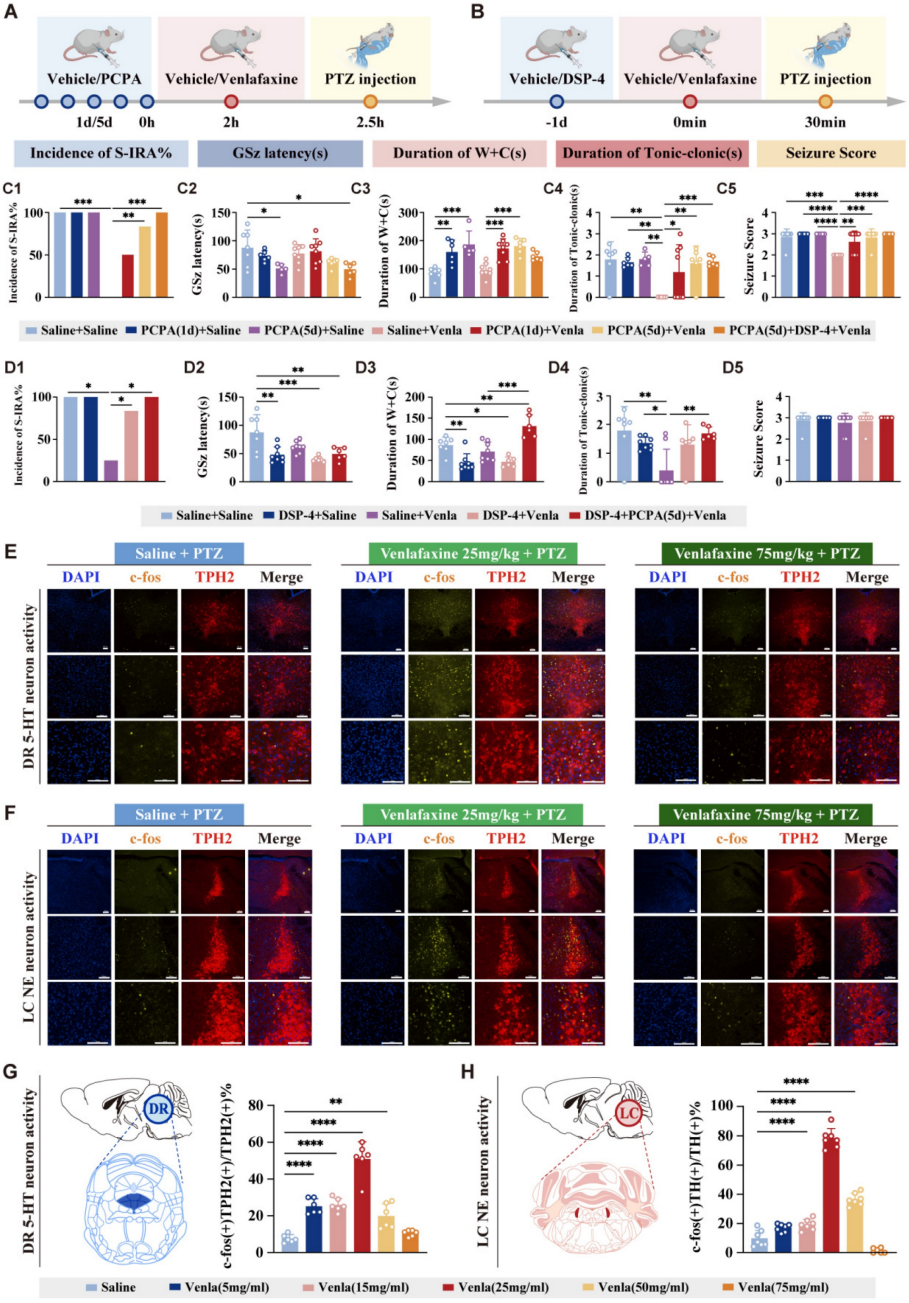
** Both DR 5-HT neurons and LC NE neurons are involved in the regulation of SUDEP.** Experimental protocol for the observations in the DBA/1 mice induced by PTZ injection following IP injection of PCPA and venlafaxine. Experimental protocol for the observations in the DBA/1 mice induced by PTZ injection following IP injection of DSP-4 and venlafaxine. **(C1-5)** The incidence of S-IRA evoked by PTZ in the group with venlafaxine was significantly reduced compared to the group with Saline, the group with PCPA (5d) + venlafaxine and the group with PCPA (5d) + DSP-4 + venlafaxine. The GSz latency in the group with PCPA (5d) and the group with PCPA (5d) + DSP-4 + venlafaxine was markedly lower than the group with Saline. The duration of wild running and clonic seizures in the group with PCPA (1d) and the group with PCPA (5d) was markedly higher than the group with Saline, and the same significant goes for the group with venlafaxine, the group with PCPA (1d) + venlafaxine and the group with PCPA (5d) + venlafaxine. The duration of tonic-clonic seizures and seizure score in the group with venlafaxine was significantly lower compared with the other groups. **(D1-5)** The incidence of S-IRA evoked by PTZ in the group with venlafaxine was significantly reduced compared to the group with Saline, the group with DSP-4 + venlafaxine and the group with DSP-4 + PCPA (5d) + venlafaxine. The GSz latency in the group with DSP-4, the group with DSP-4 + venlafaxine and the group with DSP-4 + PCPA (5d) + venlafaxine was markedly lower than the group with Saline. The duration of wild running and clonic seizures in the group with DSP-4, the group with DSP-4 + venlafaxine and the group with DSP-4 + PCPA (5d) + venlafaxine was markedly decreased than the group with Saline. The duration of wild running and clonic seizures in the group with venlafaxine was significantly decreased than the group with DSP-4 + PCPA (5d) + venlafaxine. The duration of tonic-clonic seizures in the group with venlafaxine was significantly lower compared with the group with Saline, the group with DSP-4 and the group with DSP-4 + PCPA (5d) + venlafaxine. No obvious differences were observed between treatment groups in the seizure score (*p* > 0.05). **(E)** Representative images of staining for c-fos, TPH2 and DAPI in the DR in the groups with Saline + PTZ, venlafaxine 25mg/kg + PTZ and venlafaxine 75mg/kg + PTZ. **(F)** Representative images of staining for c-fos, TH and DAPI in the LC in the groups with Saline + PTZ, venlafaxine 25mg/kg + PTZ and venlafaxine 75mg/kg + PTZ. **(G)** The quantification of c-fos (+)/TPH2 (+) section in the DR. Significantly more c-fos (+)/TPH2 (+) cells were observed in the group with 5mg/kg venlafaxine, 15mg/kg venlafaxine, 25mg/kg venlafaxine and 50mg/kg venlafaxine. **(H)** The quantification of c-fos (+)/TH (+) section in the LC. Significantly more c-fos (+)/TH (+) cells were observed in the group with 15mg/kg venlafaxine, 25mg/kg venlafaxine and 50mg/kg venlafaxine. ****P < 0.0001; ***P < 0.001; **P < 0.01; *P < 0.05; Data are mean±SEM; i.p.: intraperitoneal injection; Scale bar = 100 μm.

Based on the pharmacological properties of venlafaxine, acting on 5-HT receptors at low doses, similar to SSRI, and both 5-HT and NE receptors at medium to high doses, the PCPA (a TPH inhibitor, the rate-limiting enzyme in 5-HT synthesis) and DSP-4 (a neurotoxin targeting noradrenergic neurons) were utilized to confirm its dual action at the minimum effective dose (Fig. 4 A, B). PCPA significantly increased S-IRA incidence compared with the experimental group with venlafaxine alone (P < 0.01, Fig. 4C1). Similarly, DSP-4 also reversed venlafaxine's reduction of S-IRA (P < 0.05, Fig. 4D1). PCPA and DSP-4 completely reversed the decrease of S-IRA induced by venlafaxine (P < 0.0001, Fig. 4 C1, D1). These results suggested that venlafaxine reduced S-IRA incidence through 5-HT2A and NE-α1 receptors, and interference with either receptor reversed its efficacy.

TPH2 is a rate-limiting enzyme for the synthesis of 5-HT, and its expression level is closely related to the activity of 5-HT synthesis, and is mainly expressed in 5-HT neurons. TH is a key enzyme in the NE synthesis pathway, which catalyzes the conversion of tyrosine to dopamine, which is the first step in the NE synthesis pathway, and the expression of TH can also reflect the activity and functional status of NE neurons. TPH2 (representing 5-HT neurons) and TH (representing NE neurons) were used to track neuronal activity (Fig. 4E, F). In the PTZ model, the expression of c-fos in DR^5-HT^ and LC^NE^ neurons increased first and then decreased with the increase of venlafaxine dose.

Compared with the control group, the c-fos expression in DR^5-HT^ neurons were significantly increased at 5 mg/kg, 15 mg/kg, 25 mg/kg, and 50 mg/kg of venlafaxine, suggesting involvement in regulating SUDEP (P < 0.001, Fig. 4E, G). venlafaxine was 15 mg/kg (P < 0.01), 25 mg/kg (P < 0.001) and 50 mg/kg (P < 0.001), the expression of c-fos in NE neurons in the LC was significantly increased compared to the control group, indicating that NE neurons were significantly activated (Fig. 4F, H). These results suggest that both 5-HT neurons in DR and NE neurons in LC are involved in the mechanism of regulating the occurrence of SUDEP, and with the increase of venlafaxine dose, 5-HT neurons in DR and NE neurons in LC may be activated first and then inhibited, which may be related to positive and negative feedback caused by drugs.

These findings revealed the role of DR^5-HT^ and LC^NE^ neurons in S-IRA regulation, demonstrating the inhibition of SUDEP by the DR-LC neural pathway, especially from the peripheral pathway. However, due to the delayed nature of c-fos expression, further real-time monitoring methods were required to fully elucidate neuronal activity in this pathway.

### ICV administration of venlafaxine significantly decreased S-IRA incidence by activating DR-LC neural pathway

**Figure 5:**
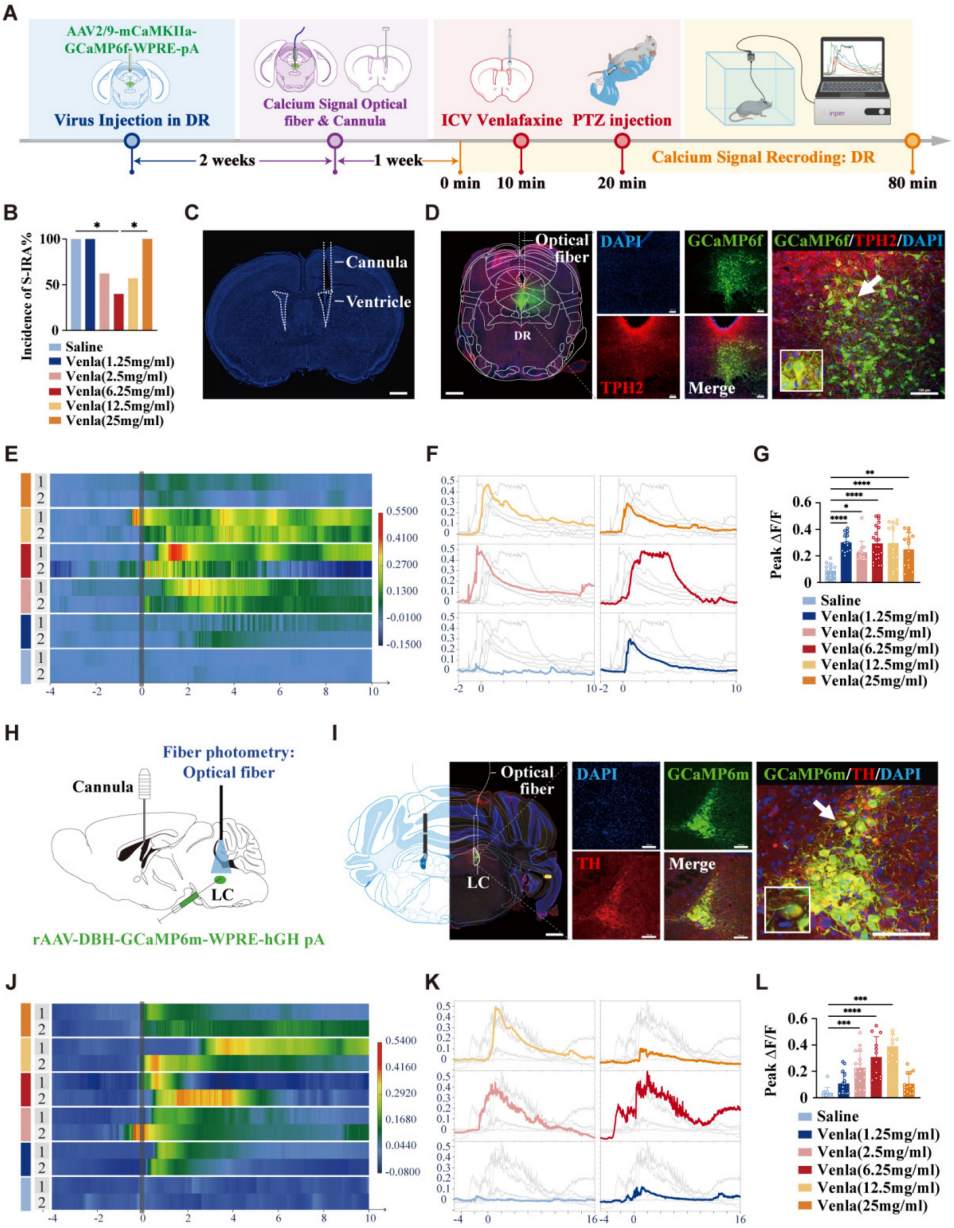
** Calcium signaling records tonic seizures in DR 5-HT neuron and LC NE neuron demonstrate the effect of reducing the incidence of S-IRA by activation of both neurons.** Experimental protocol for calcium signal recording of DR 5-HT neurons or LC NE neurons and ICV injection of venlafaxine in the PTZ-induced TH-Cre DBA/1 mice. **(B)** The incidence of S-IRA evoked by PTZ in the group with 6.25mg/ml venlafaxine was markedly reduced compared to the group with Saline and the group with 25mg/ml venlafaxine. **(C)** Representative image of the track of cannula implanted for ICV injection. **(D)** Representative images show the optical fiber location and the co-expression of GCaMP6f, TPH2 and DAPI in the DR. **(E-F)** Heatmap and statistical diagram of calcium signaling changes in DR during the observation phase from two representative mice in the groups with ICV injection of Saline or different dose of venlafaxine. **(G)** The peak ΔF/F in DR of tonic seizures was significantly increased in all groups with ICV injection of different dose of venlafaxine than in the control group. **(H)** Schematic diagram of the positions for virus injection and optic fibers implantation in the LC and ICV injection of venlafaxine. **(I)** Representative images show the optical fiber location and the co-expression of GCaMP6f, TH and DAPI in the LC. **(J-K)** Heatmap and statistical diagram of calcium signaling changes in LC during the observation phase from two representative mice in the groups with ICV injection of Saline or different dose of venlafaxine. **(L)** The peak ΔF/F in DR of tonic seizures was significantly increased in the groups with ICV injection of 2.5mg/ml venlafaxine, 6.25mg/ml venlafaxine and 12.5mg/ml venlafaxine than in the control group. ****P < 0.0001; ***P < 0.001; **P < 0.01; *P < 0.05; Data are mean±SEM; i.p.: intraperitoneal injection; icv: intracerebroventricular; Scale bar = 100 μm.

To further identify the specific target nuclei and locations within the CNS, cannulas were implanted in the lateral ventricles of mice, and calcium signaling viruses were microinjected into the DR and LC. Calcium signaling recording was performed three weeks post-infection. Ten min after recording, varying doses of venlafaxine (2.0 µL) were administered via ICV cannulas, followed by PTZ injection. Ten min later, PTZ was injected intraperitoneally, and changes in the activity of 5-HT neurons in the DR and NE neurons in the LC were recorded (Fig. 5A-C). Results showed that at a concentration of 6.25 mg/mL, venlafaxine significantly reduced S-IRA incidence compared to control (P < 0.05, Fig. 5B). However, as the concentration of venlafaxine increased, the incidence of S-IRA in mice gradually increased. When venlafaxine was administered at 25mg/ml, the incidence of S-IRA was significantly higher than that in the 6.25mg/ml group (P < 0.05, Fig. 5B). This trend was consistent with the trend of S-IRA incidence in the PTZ model when different concentrations of venlafaxine were injected intraperitoneally. During clonic seizures in mice, calcium signaling changes in DR^5-HT^ neurons increased with venlafaxine starting at 1.25 mg/mL (P < 0.01, Fig. 5E-G), peaking and then decreasing with higher concentrations (Fig. 5G). Similarly, LC^NE^ neurons e showed significant increases in calcium signaling starting at 2.5 mg/mL, peaking at 12.5 mg/mL (P < 0.001, Fig. 5H-L), and decreasing with further increases in concentration (Fig. 5L).

These results further suggest that, similar to peripheral administration, we applied the expression of calcium signal at the synaptic level to verify that after intraventricular administration, 5-HT neurons in DR and NE neurons in LC were first activated and then inhibited with the increase of venlafaxine concentration, which was consistent with the statistical trend of the incidence of S-IRA in mice.

### Optogenetic and Chemogenetic activation of the DR^5-HT^ and LC^NE^ neurons revealed the sequential order of the DR-LC neural pathway

Despite validating our conclusions on the DR-LC neural pathway's synergistic regulation of S-IRA incidence through rigorous peripheral pharmacological approaches and central calcium signaling analysis, there remains a gap in specific validation tailored specifically to this finding. Given the intimate interplay between these two entities, we endeavor to elucidate their mutual regulatory dynamics and delve into the intricate upstream and downstream sequences that govern the function of the DR^5-HT^ and LC^NE^, thereby enhancing the comprehensiveness of our understanding.

**Figure 6:**
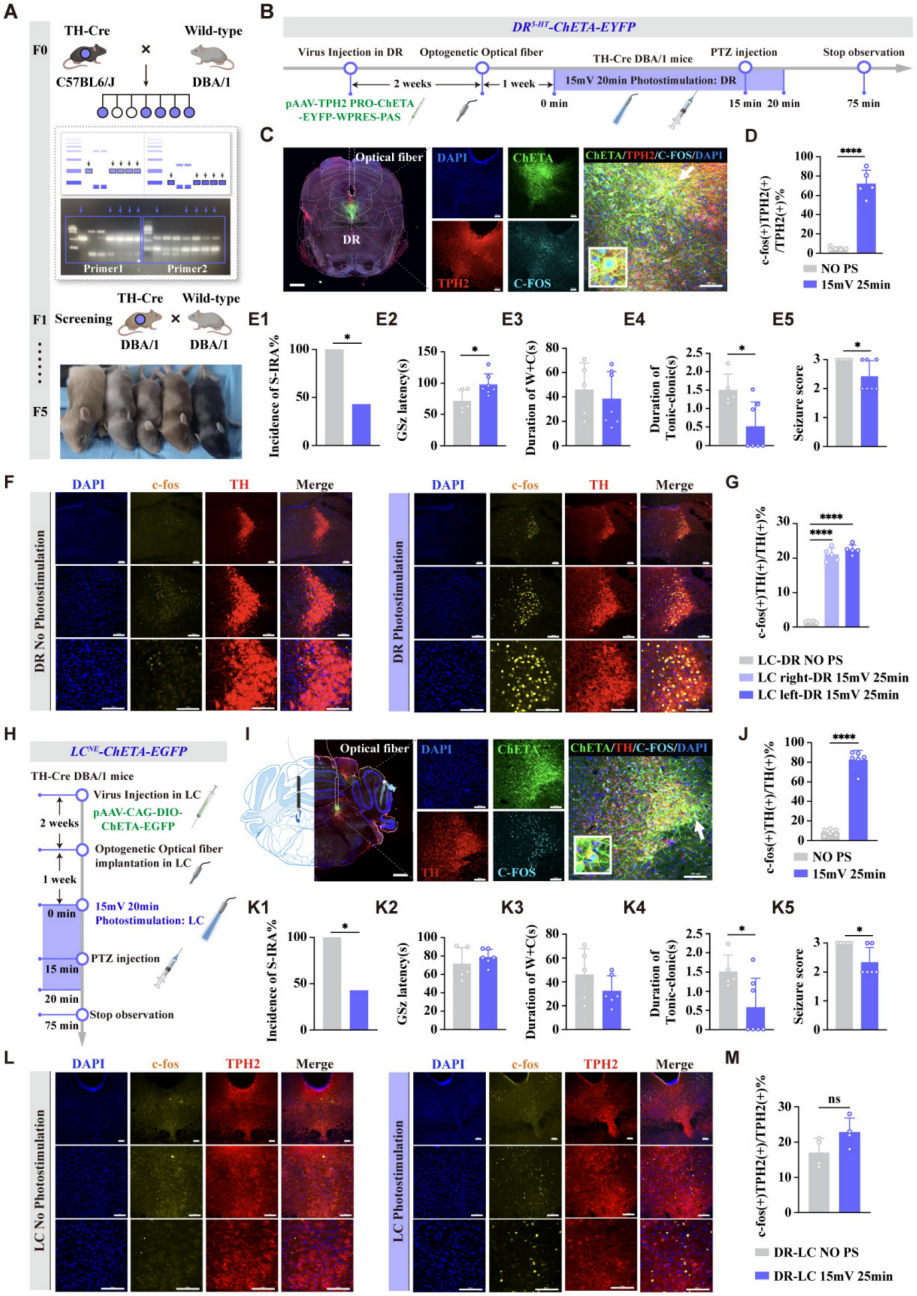
** Optogenetic activation of DR 5-HT neurons and LC NE neurons reduced the incidence of S-IRA evoked by PTZ. (A)** A diagram of breeding and genotyping TH-Cre DBA/1 mice. **(B)** Experimental protocol for optogenetic stimulation of DR 5-HT neurons in the PTZ-induced TH-Cre DBA/1 mice. **(C)** Representative images show the optical fiber location and the co-expression of CHETA, TPH2, C-FOS and DAPI in the DR. **(D)** The quantification of c-fos (+)/TPH2 (+) section in the DR with or without DR photostimulation Significantly more c-fos (+)/TPH2 (+) cells in the DR were observed in the group with photostimulation (15mv 20min). **(E1-5)** The incidence of S-IRA evoked by PTZ injection, the duration of tonic-clonic seizures and the seizure score in the group with DR photostimulation (15mv 20min) were markedly reduced compared to the group without DR photostimulation. The GSz latency in the group with DR photostimulation (15mv 20min) were markedly increased compared to the group without DR photostimulation. No obvious differences were observed between treatment groups in the duration of wild running and clonic seizures (*p* > 0.05). **(F)** Representative images of staining for C-FOS, TH, and DAPI in the LC in the groups with and without DR photostimulation. **(G)** The quantification of c-fos (+)/TH (+) section in the LC with or without DR photostimulation. Significantly more c-fos (+)/TH (+) cells in the bilateral LC were observed in the group with DR photostimulation. **(H)** Experimental protocol for optogenetic stimulation of LC NE neurons in the PTZ-induced TH-Cre DBA/1 mice. **(I)** Representative images show the optical fiber location and the co-expression of CHETA, TH, C-FOS and DAPI in the LC. **(J)** The quantification of c-fos (+)/TH (+) section in the LC with or without LC photostimulation. Significantly more c-fos (+)/TH (+) cells were observed in the group with photostimulation (15mv 25min). **(K1-5)** The incidence of S-IRA evoked by PTZ injection, the duration of tonic-clonic seizures and the seizure score in the group with LC photostimulation (15mv 20min) were markedly reduced compared to the group without LC photostimulation. No obvious differences were observed between treatment groups in the GSz latency and the duration of wild running and clonic seizures (*p* > 0.05). **(L)** Representative images of staining for C-FOS, TPH2, and DAPI in the DR in the groups with and without LC photostimulation. **(M)** The quantification of c-fos (+)/TPH2 (+) section in the DR with or without LC photostimulation. Significantly more c-fos (+)/TPH2 (+) cells were observed in the group with LC photostimulation. ****P < 0.0001; *P < 0.05; ns: P > 0.05; Data are mean±SEM; PS: photostimulation; NO PS: no photostimulation; i.p.: intraperitoneal injection; Scale bar = 100 μm.

To investigate the specific roles and interconnections of DR^5-HT^ and LC^NE^ neurons in SUDEP and the connection between them, optogenetic viruses were injected into the DR, and after three weeks, light stimulation was applied (Fig. 6A-D). The results showed that activating DR^5-HT^ neurons with light stimulation significantly reduced the incidence of S-IRA, prolonged the latency of epilepsy, shortened the duration of tonic spasms, and decreased the epilepsy score (*P* < 0.05, Fig. 6E1-E5). Meanwhile, it was also increased the expression of c-fos in NE neurons LC^NE^ neurons (*P* < 0.001, Fig. 6F, G). This indicated that activating DR^5-HT^ neurons can significantly reduce the incidence of S-IRA and also activate LC^NE^ neurons.

In a separate experiment, optogenetic viruses were injected into the bilateral LC of TH-cre transgenic DBA/1 background mice (Fig. 6H-J). The results showed that light stimulation activated NE neurons in the bilateral LC. Compared with the non-light stimulation group, the incidence of S-IRA in the light stimulation group was significantly reduced, and the duration of tonic convulsion was also significantly shortened, resulting in a lower epilepsy score (*P* < 0.05, Fig. 6K1, K4, K5). However, there were no significant differences in the latency of GSz onset and the duration of running convulsion (*P* > 0.05, Fig. 6K2, K3). Compared with the non-light stimulation group, Light stimulation of the expression of LC^NE^ neurons did not significantly increase the c-fos expression in DR^5-HT^ neurons (*P* > 0.05, Fig. 6L, M). These results indicated that optogenetic activation of LC^NE^ neurons could significantly reduce the incidence of S-IRA but could not activate DR^5-HT^ neurons.

**Figure 7:**
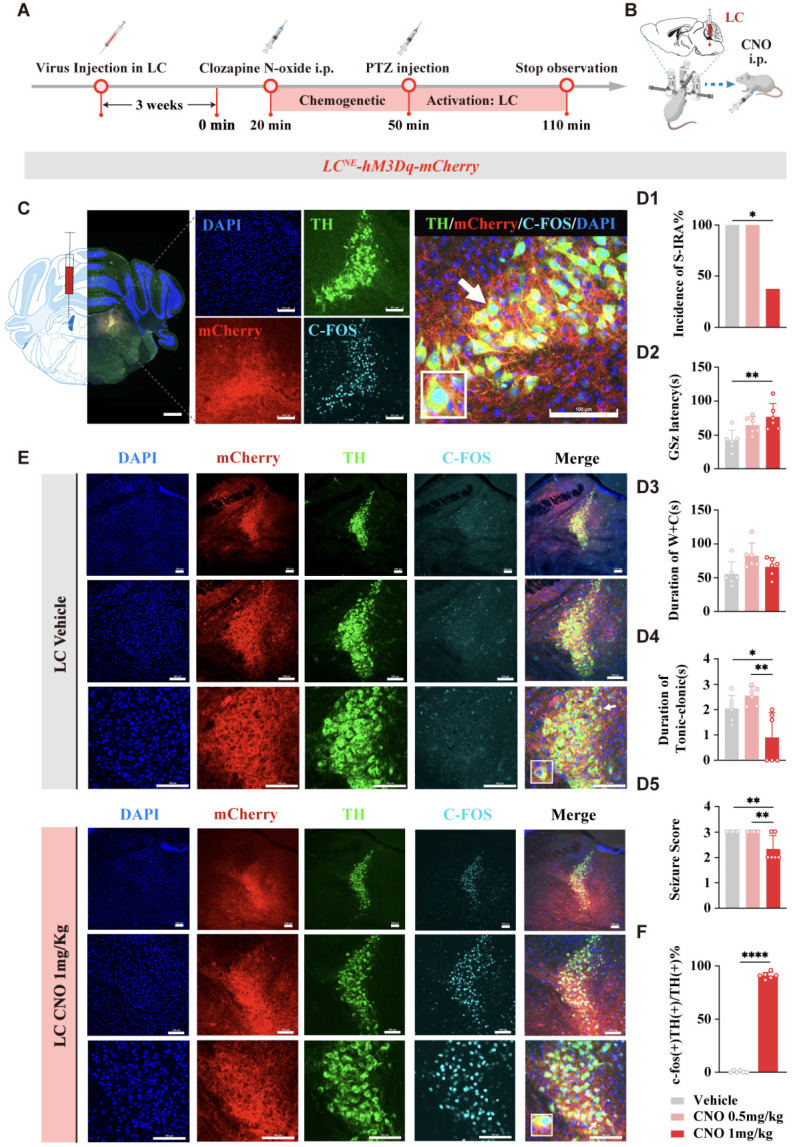
** Chemogenetic activation of LC NE neurons significantly reduced the incidence of S-IRA evoked by PTZ. (A)** Experimental protocol for chemogenetic activation of LC NE neurons in the PTZ-induced DBA/1 mice. **(B)** Schematic diagram of the location of virus (mTH-Cre-AAV+AAV-EF1a-DIO-hM3Dq-mCherry) injection in LC and IP injection of CNO. **(C)** Representative images show the location of virus injection and the co-expression of TH, mCherry, C-FOS and DAPI in the LC**. (D1-5)** The incidence of S-IRA evoked by PTZ in the group with 1mg/kg CNO was markedly reduced compared to the group with Vehicle. The GSz latency in the group with 1mg/kg CNO was significantly higher than the group with Vehicle. The duration of tonic-clonic seizures and the seizure score in the group with 1mg/kg CNO was significantly lower than the group with Vehicle and the group with 0.5mg/kg CNO. No obvious differences were observed between treatment groups in the duration of wild running and clonic seizures (*p* > 0.05). **(E)** Representative images show the co-expression and staining for TH, mCherry, C-FOS and DAPI in the LC with or without CNO activation. **(F)** The quantification of c-fos (+)/TH (+) section in the LC with or without CNO activation. Significantly more c-fos (+)/TH (+) cells were observed in the 1mg/kg CNO group than in the Vehicle group (*p* < 0.0001). ****P < 0.0001; **P < 0.01; *P < 0.05; Data are mean±SEM; i.p.: intraperitoneal injection; Scale bar = 100 μm.

To further investigate the specific roles of DR^5-HT^ neurons and LC^NE^ neurons in SUDEP and the connection between them, we injected chemogenetic viruses into the bilateral LC of TH-cre transgenic DBA/1 background mice. Following three weeks of infection, chemogenetic experiments were conducted. PTZ was injected 30 min after IP injection of different doses of CNO. Mice behaviors were observed and recorded, and immunohistochemical analysis was performed to quantify c-fos expression in TH neurons within the LC of mice (Fig. 7A-C). The results showed that chemogenetic activation of NE neurons in the bilateral LC significantly reduced the incidence of S-IRA in the 1 mg/kg CNO group compared to the saline group (*P* < 0.05), while there was no significant difference in the 0.5 mg/kg CNO group compared to the saline group (*P* > 0.05, Fig. 7D1).

In addition, we found that compared with the control group, IP injection of 1 mg/kg CNO prolonged the latency of epileptic seizures, shortened the duration of tonic convulsions, and reduced the epilepsy score (Fig. 7D2, D4, D5). Furthermore, CNO treatment significantly increased the expression of c-fos in LC^NE^ neurons (*P* > 0.05, Fig. 7E, F). These suggested that chemogenetic activation of LC^NE^ neurons at a certain intensity can significantly reduce the incidence of S-IRA and markedly activate LC^NE^ neurons.

### The projection relationship of the DR-LC-PBC neural pathway established by bidirectional verification

**Figure 8:**
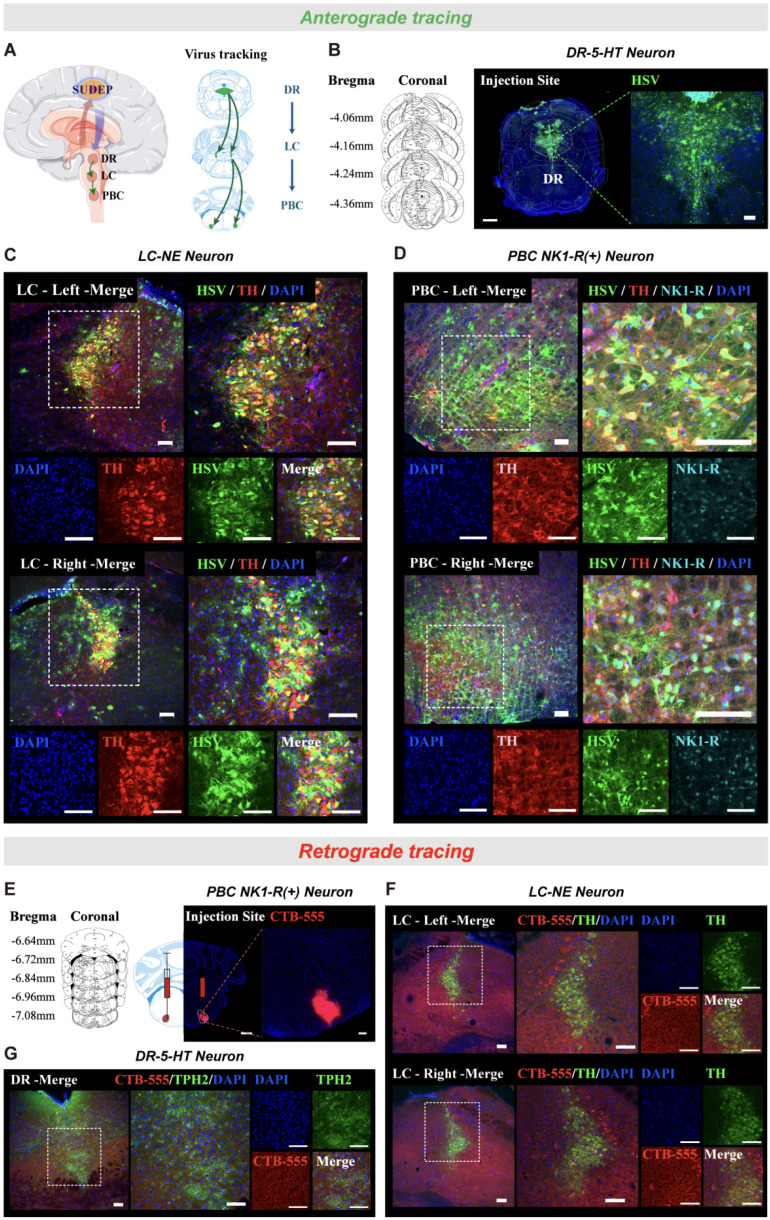
** The existence of projection relationship of DR**"**LC**"**PBC neural pathway by anterograde tracing. (A)** Experimental protocol for the anterograde tracing by injecting anterograde tracing virus HSV into DR. **(B)** Representative coronal brain slice, showing the location of HSV injected in the DR. **(C)** Representative images show the co-expression and staining of HSV, TH and DAPI in the bilateral LC. **(D)** Representative images show the co-expression and staining of HSV, TH, NK1-R and DAPI in the bilateral PBC. **(E)** Representative coronal brain slice, showing the location of CTB-555 injected in the PBC. **(F)** Representative images show the co-expression and staining of CTB-555, TH and DAPI in the bilateral LC. **(G)** Representative images show the co-expression and staining of CTB-555, TPH2 and DAPI in the DR. Scale bar = 100 μm.

The above results suggested that optogenetic activation of DR^5-HT^ neurons can significantly reduce the incidence of S-IRA and activate LC^NE^ neurons. Similarly, optogenetic and chemogenetic activation of LC^NE^ neurons also significantly reduced the incidence of S-IRA, but did not activate DR^5-HT^ neurons. Considering both nuclei are involved in monoaminergic neurotransmitter production but do not directly regulate respiration, we hypothesized that they exert influence through interconnected pathways. Based on previous research, activating DR^5-HT^ neurons can reduce the incidence of S-IRA through the 5-HT2A receptor in PBC, which plays a key role in generating respiratory rhythms. While PBC neurons don't directly project to phrenic motor neurons, they coordinate respiratory activity by interacting with neurons in other brainstem regions. Since the LC projects to premotor respiratory neurons, it might influence respiratory rhythms through this interaction. Therefore, we hypothesized a projection relationship between the DR, LC, and PBC, where DR^5-HT^ neurons act as upstream regulators of LC^NE^ neurons, which then modulate PBC activity. To test this hypothesis, we microinjected HSV virus into the DR for anterograde tracing (Fig. 8A-C). Immunohistochemical analysis showed co-localization of HSV and TH in both the LC and PBC, confirming neural projections between these regions (n = 5, Fig. 8C, D).

For further validation, we performed retrograde tracing by injecting CTB virus into the PBC and observed labeling in LC^NE^ neurons and DR^5-HT^ neurons (Fig. 8E). CTB was significantly labeled in DR and LC, and the fluorescence expression increased significantly (Fig. 8F, G). The results confirmed that LC^NE^ neurons are upstream of PBC neurons, with DR^5-HT^ neurons acting as upstream regulators of the LC, thus modulating PBC activity. These findings suggested that DR 5-HT neurons may reduce the occurrence of S-IRA by regulating PBC activity via LC^NE^ neurons.

### Chemogenetic activation of the LC^NE^ significantly enhanced the PBC^NE^ activity

**Figure 9:**
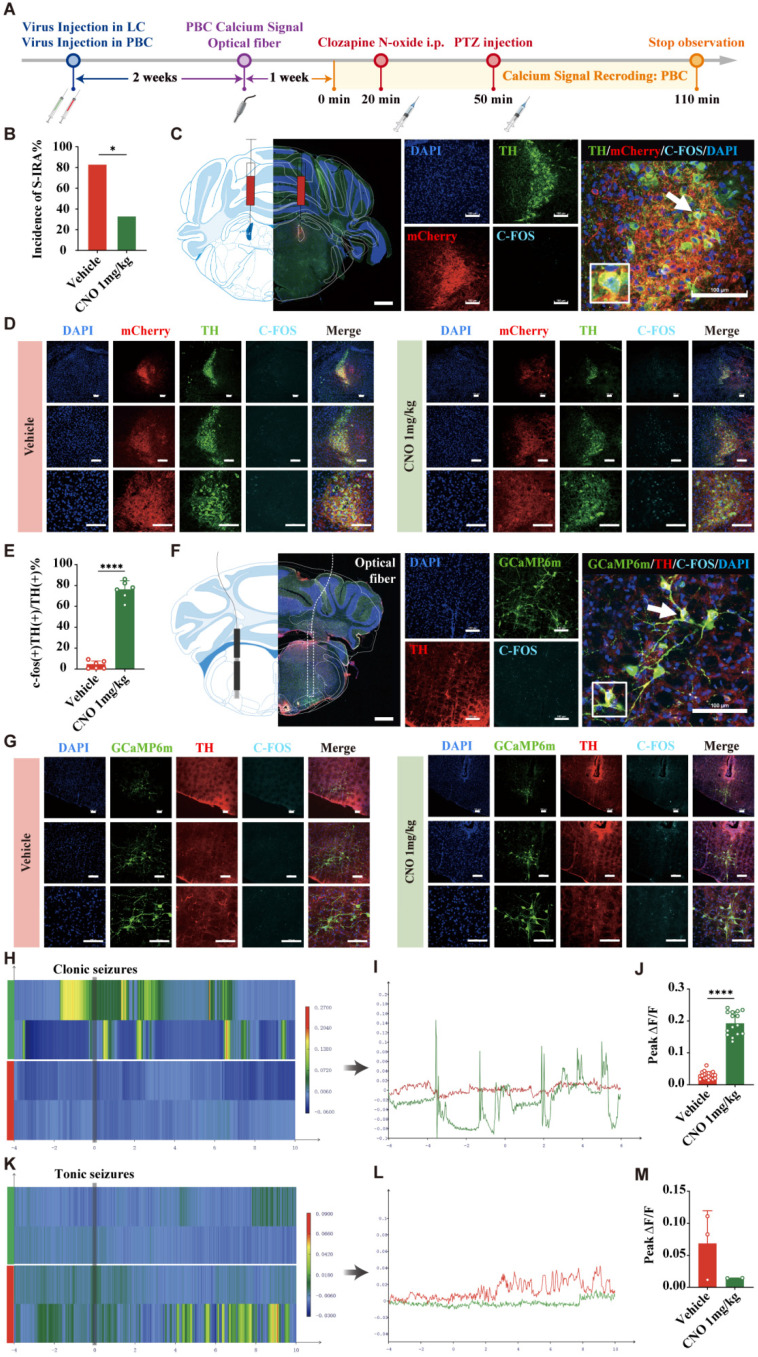
** Chemogenetic activation of LC NE neurons attenuated the occurance of S-IRA evoked by PTZ and activated PBC NE neurons.** Experimental protocol for chemogenetic activation of LC NE neurons and recording the calcium signalng of PBC in the PTZ-induced DBA/1 mice. The incidence of S-IRA evoked by PTZ in the group with 1mg/kg CNO was markedly reduced compared to the group with Vehicle. **(C)** Representative images show the location of virus injection and the co-expression of TH, mCherry, C-FOS and DAPI in the LC. Scale bar = 100 μm. **(D)** Representative images show the co-expression and staining for TH, mCherry, C-FOS and DAPI in the LC with or without CNO treatment. Scale bar = 100 μm. **(E)** The quantification of c-fos (+)/TH (+) section in the LC with or without chemogenetic activation. **(F)** Representative images show the location of virus injection and the co-expression of GCaMP6m, TH, C-FOS and DAPI in the LC. Scale bar = 100 μm. **(G)** Representative images show the co-expression and staining for GCaMP6m, TH, C-FOS and DAPI in the LC with or without CNO treatment. Scale bar = 100 μm. **(H-I)** Heatmap and statistical diagram of calcium signaling changes in PBC during the observation phase from two representative mice in the groups with ICV injection of Saline or 1 mg/kg CNO during clonic seizures. **(G)** The quantification of peak ΔF/F in PBC during clonic seizures. **(K-L)** Heatmap and statistical diagram of calcium signaling changes in PBC during the observation phase from two representative mice in the groups with ICV injection of Saline or 1 mg/kg CNO during tonic seizures. **(M)** The quantification of peak ΔF/F in PBC during tonic seizures.

To further investigate the role of activation of the LC^NE^ neurons on the PBC^NE^ neurons and and its impact on on SUDEP, LC^NE^ neurons were stimulated by 1 mg/kg CNO, and the incidence of S-IRA, c-fos expression, and calcium signaling changes in PBC NE neurons were measured (Fig. 9A). Our findings revealed that 1 mg/kg CNO significantly reduced the incidence of S-IRA induced by PTZ, compared to the vehicle group (*P* < 0.05, Fig. 9B). The c-fos expression of LC^NE^ neurons in the 1mg/kg CNO group was higher than in the vehicle group (*P* < 0.001, Fig. 9C-E). This increase in c-fos expression was also evident in PBC^NE^ neurons (Fig. 9F, G). Furthermore, calcium signaling in PBC^NE^ neurons was significantly elevated during clonic seizures following 1 mg/kg CNO administration (*P* < 0.001, Fig. 9H-J). However, no significant difference was observed during tonic seizures (*P* > 0.05, Fig. 9K-M). These results suggested that activating LC^NE^ neurons attenuated the occurrence of S-IRA by activating PBC^NE^ neurons.

### The DR-LC-PBC relied on LC^NE^ neurons to regulate S-IRA synergistically

**Figure 10:**
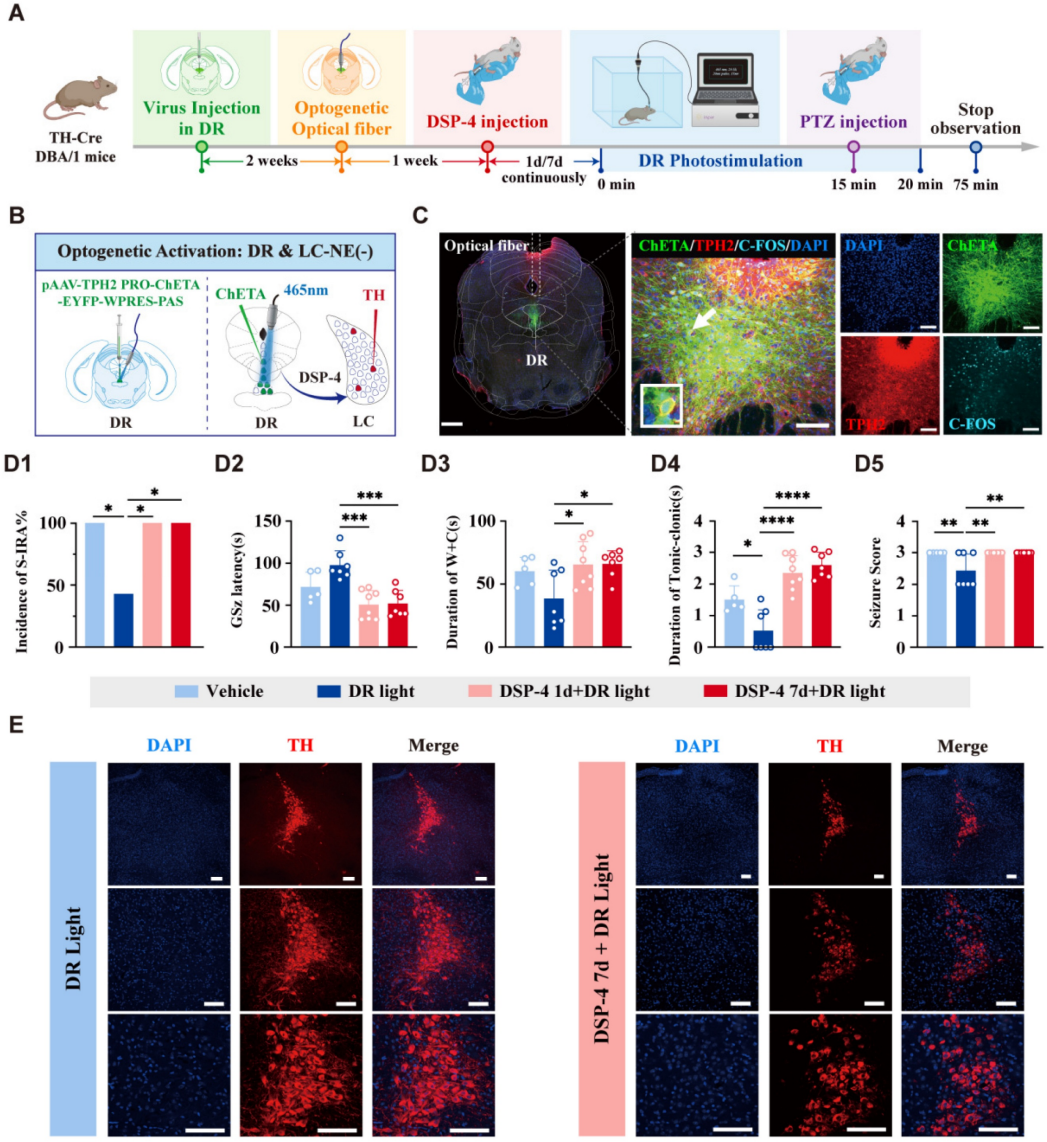
**Optogenetic activation of DR 5-HT neurons reduced the incidence of S-IRA evoked by PTZ, and DSP-4 which degraded central LC NE neurons can significantly reverse the effect. (A)** Experimental protocol for optogenetic activation of DR 5-HT neurons and IP injection of DSP-4 in the PTZ-induced TH-Cre DBA/1 mice. **(B)** Schematic diagram of the positions for the virus (pAAV-TPH2 PRO-ChETA-EYFP-WPRES-PAS) injection and optic fibers implantation in DR. **(C)** Representative images show the optical fiber location and the co-expression of ChETA, C-FOS, TPH2 and DAPI in the DR. **(D1-5)** The incidence of S-IRA evoked by PTZ, the duration of tonic-clonic seizures and the seizure score in the group with DR light was markedly reduced compared to the group with Vehicle, the group with DSP-4 1d + DR light and the group with DSP-4 7d + DR light. The GSz latency in the group with DR light was significantly higher than the group with DSP-4 1d + DR light and the group with DSP-4 7d + DR light. The duration of wild running and clonic seizures in the group with DR light was markedly lower than the group with DSP-4 1d + DR light and the group with DSP-4 7d + DR light. **(E)** Representative images of staining for TH and DAPI in the LC in the groups with DR light and the group with DSP-4 7d + DR light. ****P < 0.0001; ***P < 0.001; **P < 0.01; *P < 0.05; Data are mean±SEM; DR Light: DR photostimulation; i.p.: intraperitoneal injection; Scale bar = 100 μm.

Above results have shown that DR^5-HT^-LC^NE^-PBC^NE^ neural circuit can reduce the incidence of S-IRA, while previous research results have shown that activating the DR-PBC neural circuit has effectively inhibited the S-IRA, but the significance of the LC as a relay station remains unclear. To further verify whether the DR and LC exhibit an interdependent relationship in jointly regulating SUDEP, we delivered virus and optical fibers into the DR and was administered DSP-4intraperitoneally for 1 or 7 days (Fig. 9A-C). The results showed that optogenetic activation of DR^5-HT^ neurons significantly reduced the incidence of S-IRA. However, this inhibitory effect was reversed by DSP-4 (Fig. 10D1-D5). Notably, DSP-4 treatment for 7 days led to a significant reduction in LC^NE^ neurons (Fig. 10D1-D5). These results suggested that activation of DR^5-HT^ neurons, which depends on the LC^NE^ neurons, exerted its effect in reducing the incidence of S-IRA. Byword, the 5-HTergic and NEergic systems intrinsically regulate the SUDEP in a synergistic-dependent manner.

### DR-LC-PBC neural circuit collectively regulates SUDEP in a synergistic-dependent manner through the cooperation of central 5-HTergic and NEergic systems by 5-HT2A and NE-α1 receptors

**Figure 11:**
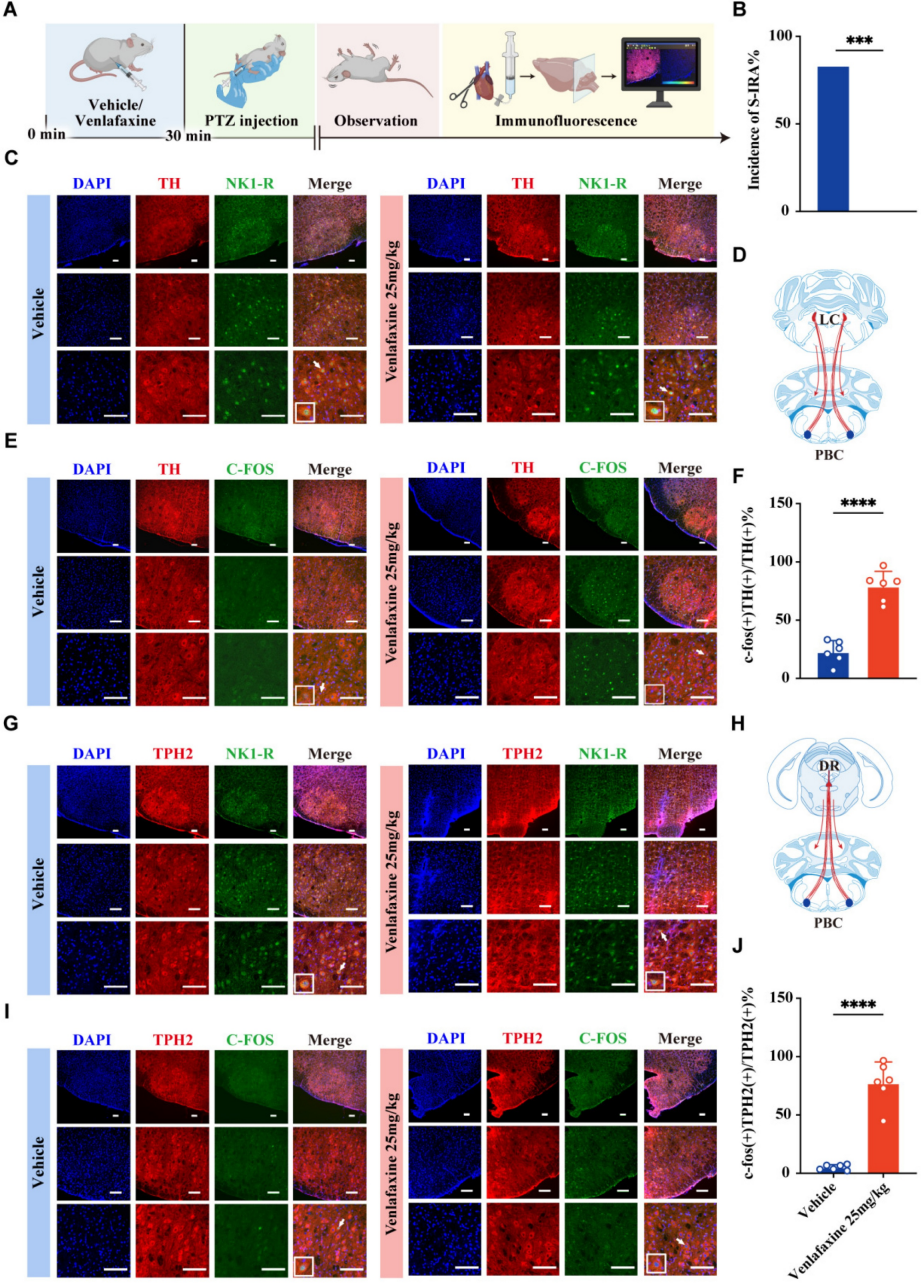
**Pharmacological activation of DR and LC activated the TPH2 and NE neurons in PBC. (A)** Experimental protocol for activation of DR and LC, observation of behaviors, and immunofluorescence. **(B)** The incidence of S-IRA evoked by PTZ in the group with activation of DR and LC was reduced compared to the group with saline. **(C)** Representative images show the co-expression and staining for TH, NK1-R and DAPI in PBC with or without activation of DR and LC. Scale bar = 100 μm. **(D)** Schematic diagram of the regulation of LC to PBC. **(E)** Representative images show the co-expression and staining for TH, c-fos and DAPI in PBC with or without activation of DR and LC. Scale bar = 100 μm. **(F)** The quantification of c-fos (+)/TH (+) section in the PBC. **(G)** Representative images show the co-expression and staining for TPH2, NK1-R and DAPI in PBC with or without activation of DR and LC. Scale bar = 100 μm. **(H)** Schematic diagram of the regulation of LC to PBC. **(I)** Representative images show the co-expression and staining for TPH2, c-fos and DAPI in PBC with or without activation of DR and LC. Scale bar = 100 μm. **(J)** The quantification of c-fos (+)/TPH2 (+) section in the PBC.

To further verify the regulation of PBC by DR and LC, we conducted pharmacological activation experiments to observe the effects on PBC^5-HT^ and PBC^NE^ neurons (Fig. 11A). Consistent with the previous results (Fig. 3B1), activation of DR and LC reduced the incidence of S-IRA (*P* < 0.01, Fig. 11B). In addition, activating DR and LC by pharmacology enhanced the c-fos expression in PBC^NE^ neurons (*P* < 0.001, Fig. 11C-F) and PBC^5-HT^ neurons in PBC (*P* < 0.001, Fig. 11G-J).

**Figure 12:**
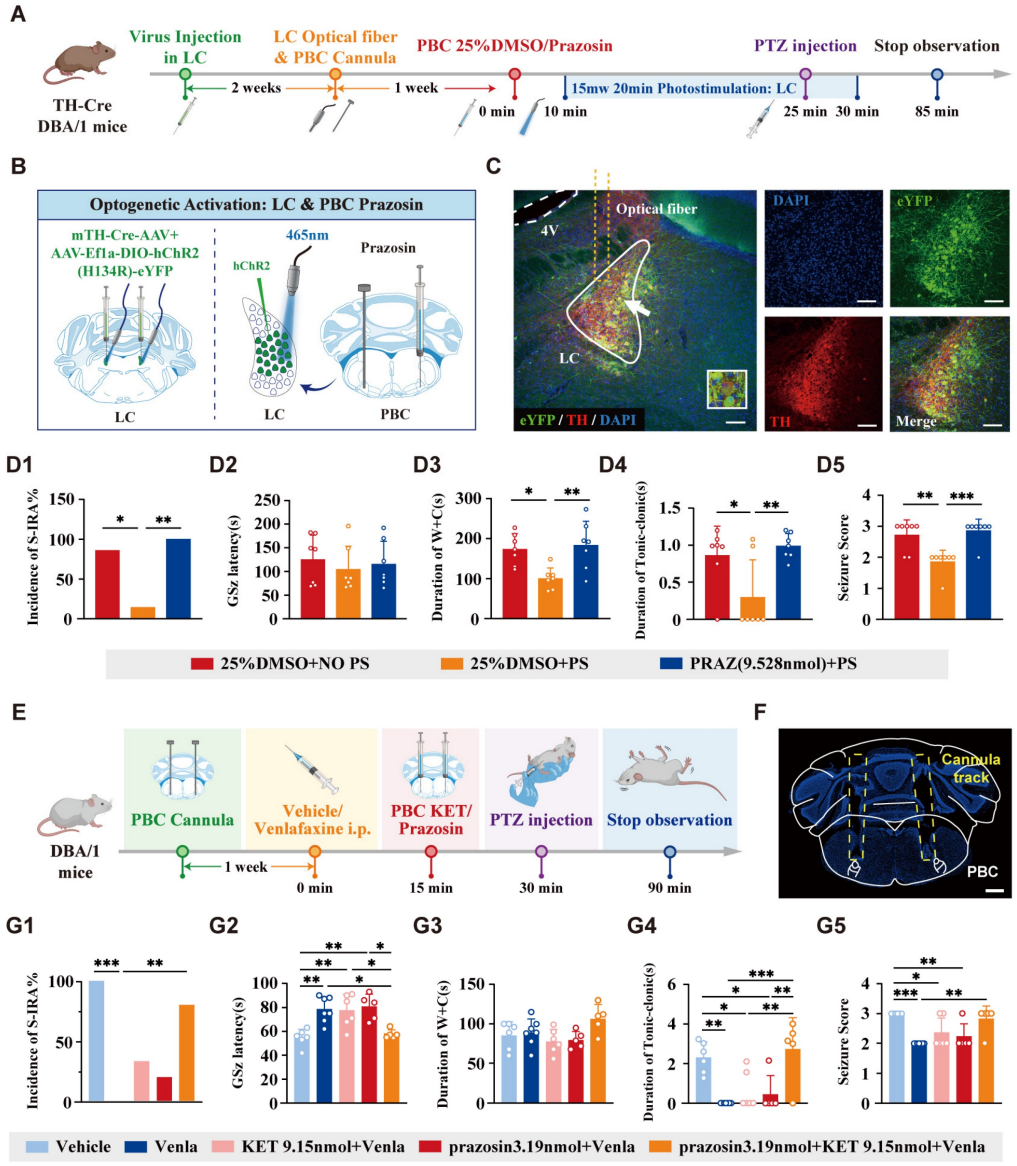
**The inhibitory effects of activation of DR-LC-PBC neural circuit can be reversed by 5-HT2A and NE-α1R antagonists microinjected in PBC. (A)** Experimental protocol for optogenetic activation of LC NE neurons and microinjection of NE-α1 receptor antagonist prazosin into bilateral PBC in the PTZ-induced TH-Cre DBA/1 mice. **(B)** Schematic diagram of the positions for virus (mTH-Cre-AAV+AAV-EF1a-DIO-hChR2(H134R)-eYFP) injection and optic fibers in the bilateral LC and prazosin microinjection into the bilateral PBC. **(C)** Representative images show the optical fiber locations and the co-expression of eYFP and TH in the LC. **(D1-5)** The incidence of S-IRA evoked by PTZ, the duration of wild running and clonic seizure or tonic-clonic seizures in the group with 25% DMSO + PS was significantly reduced compared to the group with 25% DMSO + NO PS and the group with PRAZ + PS. The seizure score in the group with 25% DMSO + PS was significantly lower compared to the group with 25% DMSO + NO PS and the group with PRAZ + PS. No obvious differences were observed between treatment groups in the GSz latency (*p* > 0.05). **(E)** Experimental protocol for embedding the cannulas in the bilateral PBC, IP injection of venlafaxine, and microinjecting 5-HT2A receptor or NE-α1R antagonists into the bilateral PBC in the PTZ-induced DBA/1 mice. **(F)** Representative image shows the tracks of cannulas implanted into the bilateral PBC. **(G1-5)** The incidence of S-IRA evoked by PTZ in the group with venlafaxine was significantly reduced compared to the group with Vehicle and the group with prazosin 3.19nmol + KET 9.15nmol + venlafaxine. The GSz latency in the group with venlafaxine, the group with KET 9.15nmol + venlafaxine and the group with prazosin 3.19nmol + venlafaxine was markedly higher than the group with Vehicle and the group with prazosin 3.19nmol + KET 9.15nmol + venlafaxine. The duration of tonic-clonic seizures in the group with venlafaxine, the group with KET 9.15nmol + venlafaxine and the group with prazosin 3.19nmol + venlafaxine was markedly lower than the group with Vehicle, and the same significant goes for the group with venlafaxine, the group with KET 9.15nmol + venlafaxine and the group with prazosin 3.19nmol + venlafaxine. The seizure score in the group with venlafaxine, the group with KET 9.15nmol + venlafaxine and the group with prazosin 3.19nmol + venlafaxine was markedly lower than the group with Vehicle. The seizure score in the group with venlafaxine was significantly reduced compared to the group with the group with prazosin 3.19nmol + KET 9.15nmol + venlafaxine. No obvious differences were observed between treatment groups in the duration of wild running and clonic seizures (*p* > 0.05). ***P < 0.001; **P < 0.01; *P < 0.05; Data are mean±SEM; PS: photostimulation; NO PS: no photostimulation; PRAZ: prazosin; i.p.: intraperitoneal inject; Scale bar = 100 μm.

**Figure 13:**
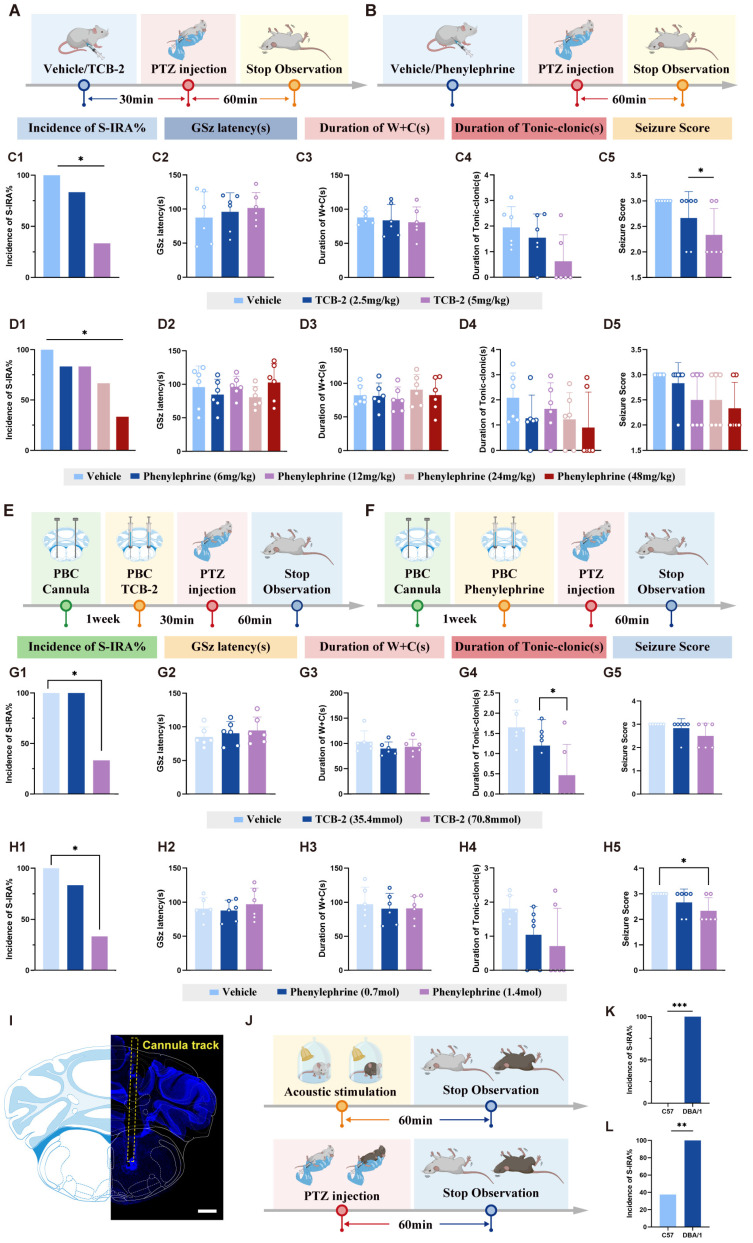
** The activation of DR-LC-PBC neural circuit can reduce the incidence of SUDEP by 5-HT2A and NE-α1R agonists microinjected in PBC. (A-B)** Experimental protocol for the observations in the DBA/1 mice induced by PTZ injection following IP injection of TCB-2 and Phenylephrine. **(C1-5)** The incidence of S-IRA evoked by PTZ in the group with TCB-2 (5mg/kg) was significantly reduced compared to the group with Vehicle. The seizure score in the group with TCB-2 (5mg/kg) was markedly lower than the group with Vehicle. No obvious differences were observed between treatment groups in the GSz latency, the duration of W+C and the duration of tonic-clonic seizures. **(D1-5)** The incidence of S-IRA evoked by PTZ in the group with Phenylephrine (48mg/kg) was significantly reduced compared to the group with Vehicle. No obvious differences were observed between treatment groups in the GSz latency, the duration of W+C, the duration of tonic-clonic seizures and seizure score. **(E-F)** Experimental protocol for embedding the cannulas in the bilateral PBC, and microinjecting 5-HT2A receptor or NE-α1R agonists into the bilateral PBC in the PTZ-induced DBA/1 mice. **(G1-5)** The incidence of S-IRA evoked by PTZ in the group with TCB-2 (70.8mmol) was significantly reduced compared to the group with Vehicle. The the duration of tonic-clonic seizures in the group with TCB-2 (70.8mmol) was markedly lower than the group with Vehicle. No obvious differences were observed between treatment groups in the GSz latency, the duration of W+C and the duration of seizure score. **(H1-5)** The incidence of S-IRA evoked by PTZ in the group with Phenylephrine (1.4mol) was significantly reduced compared to the group with Vehicle. The seizure score in the group with Phenylephrine (1.4mol) was markedly lower than the group with Vehicle. No obvious differences were observed between treatment groups in the GSz latency, the duration of W+C and the duration of tonic-clonic seizures. **(I)** Representative image shows the tracks of cannulas implanted into the bilateral PBC. **(J)** Experimental protocol for the observations in the DBA/1 and C57 mice induced by PTZ injection and acoustic stimulation. **(K)** The incidence of SUDEP in DBA/1 mice and C57 mice induced by acoustic stimulation. **(L)** The incidence of SUDEP in DBA/1 mice and C57 mice induced by PTZ injection. ***P < 0.001; **P < 0.01; *P < 0.05; Data are mean±SEM; Scale bar = 100 μm.

To identify the specific neural projections and receptors involved in these effects, we combined optogenetics with pharmacological techniques. Prazosin, an NE-α1R antagonist, was microinjecteinto the PBC and the bilateral LC was activated through photostimulation to assess the effects on SUDEP (Fig. 12A).

AAV-EF1a-DIO-hChR2 (H134R)-eYFP was microinjected into the bilateral LC and optical fibers were implanted, with drug-delivery cannulas placed in the bilateral PBC (Fig. 12B-C). The results showed that photostimulation of LC^NE^ neurons significantly reduced the incidence of S-IRA induced by PTZ compared to control groups. However, this effect was reversed by microinjection of prazosin into the PBC (Fig. 12D1). Similar trends were observed in the duration of wild running, the duration of clonic seizure or tonic-clonic seizures and seizure score (Fig. 12D3-D5).

To further verify the role of 5-HT2A and NE-α1 receptors in regulating S-IRA, cannulas were embedded in the bilateral PBC and antagonists for 5-HT2AR or NE-α1R were microinjected into the PBC following IP injection of venlafaxine (Fig. 12E-F). We found that microinjection of a 5-HT2AR or NE-α1R antagonists into the PBC reversed the pro-arousal effect of venlafaxine, and the inhibitory effect of venlafaxine on S-IRA was almost completely reversed with simultaneously administration of KET and prazosin (Fig. 12G1-G5).

Finally, to determine whether receptor activation within the PBC is sufficient to suppress SUDEP, we first performed systemic intraperitoneal injections of the 5-HT2AR agonist TCB-2 and the NE-α1R agonist phenylephrine in DBA/1 mice. Systemic administration of either agonist significantly lowered SUDEP incidence. To establish greater precision and pathway specificity, we then directly microinjected TCB-2 and phenylephrine into the PBC. Both agents markedly reduced S-IRA incidence, and notably, intra-PBC administration of TCB-2 not only decreased S-IRA occurrence but also significantly shortened the duration of tonic-clonic seizures.

These results further support the involvement of 5-HT2A and NE-α1 receptors in the regulation of SUDEP through the synergistic-dependent cooperation of central 5-HTergic and NEergic systems.

## Discussion

In this study, we established SUDEP models induced by acoustic stimulation and PTZ injection, respectively. By increasing the levels of 5-HT and NE through peripheral administration, we found that activating both the 5-HT and NE nervous systems could reduce the incidence of SUDEP, and that there is a synergistic effect between them. In the regulation of SUDEP occurrence, the 5-HT and NE systems are mutually required. Subsequently, we explored the central nervous mechanisms of SUDEP occurrence using immunofluorescence staining, calcium signal recording, optogenetic techniques, and other methods, which fully clarified that activating 5-HT neurons in the DR and NE neurons in the LC could prevent the occurrence of SUDEP, and their downstream targe is PBC, a nucleus regulating respiratory rhythms. Furthermore, using anterograde and retrograde tracing techniques, we confirmed that the LC is an important downstream target of the DRN, and 5-HT neurons within the DRN may reduce the incidence of S-IRA and SUDEP by activating NE neurons within the LC. Concurrently, it was observed that the PBC, a pivotal nucleus in the regulation of respiratory rhythm and positioned downstream of the DR and the LC, received information from these nuclei via the 5-HT2A receptor and the NE-α1 receptor, respectively. This neural communication plays a critical role in modulating the occurrence of S-IRA and SUDEP. This study primarily elucidates the potential neural mechanisms underlying the occurrence of SUDEP, thereby providing new research directions for the prevention of SUDEP.

SUDEP is the leading cause of mortality among epilepsy patients and a pressing public health concern. Research on the mechanisms and potential contributing factors of SUDEP has progressed, due to the complexity of SUDEP and the difficulty in obtaining real-time clinical data, the pathophysiological mechanisms remain unclear. As previous studies have shown, exogenously increasing 5-HT levels in DBA/1 mice and optogenetic activating 5-HT neurons in the DR can significantly reduce the incidence of S-IRA in mice, and there is a neural circuit connection with the PBC, which regulates respiratory rhythm 12,28,36. It has also been found that the NE reuptake inhibitor atomoxetine can significantly reduce the incidence of S-IRA in DBA/1 mice by increasing NE content in the brain 18,22. Considering the 5-HT and NE systems as important members of the monoaminergic neurotransmitter family, we hypothesized that there is an internal regulatory manner the 5-HT and NE systems that jointly regulates SUDEP. However, the specific regulatory roles, target sites, and whether there is a temporal relationship between the two systems remain unclear. Therefore, we designed a series of studies using pharmacology, fiber photometry, optogenetics, and chemogenetics as shown in Fig. 1.

Firstly, to explore the role of the 5-HT and NE systems in the pathogenesis of SUDEP, 5-HTP or atomoxetine were injected respectively to increase the levels of 5-HT or NE. We observed that only higher doses of 5-HTP (100-125 mg/kg) or atomoxetine (15-20 mg/kg) significantly reduced S-IRA incidence, indicating a dose-dependent effect. Nevertheless, at higher effective doses of 5-HTP or atomoxetine, although the incidence of S-IRA in mice was reduced, adverse effects such as diarrhea and poor motility were observed, which may be related to the toxic reactions caused by excessive drug doses 37. To overcome these limitations, we innovatively combined subtherapeutic low doses of both 5-HTP and atomoxetine, which together produced a robust and complete reduction in S-IRA incidence induced by acoustic stimulation and PTZ, without observable side effects. This synergistic effect also improved seizure parameters, including prolonged seizure latency and reduced seizure severity, surpassing the efficacy of either agent alone. Similar to our findings, previous studies have shown that 5-HTP, at specific dosages, can effectively reduce incidence of seizure, decrease frequency, and prolong latency. Moreover, 5-HT levels are intimately linked to the seizure threshold; lowering 5-HT levels diminishes the threshold, thereby exacerbating generalized seizure activity 38-40. Concurrently, endogenous NE exhibits anticonvulsant properties in epilepsy. Furthermore, decreased NE levels are correlated with heightened susceptibility to neuronal damage in regions marginally free of seizures and/or prone to audiogenic seizures 41. The neuroprotective effects of NE are ascribed to its role in counteracting epileptogenic circuit formation and modulating epilepsy-associated neuronal alterations, which collectively reduce central nervous system susceptibility to epileptiform activities 40. It can be inferred that the combined use of 5-HTP and Atomoxetine, which simultaneously elevate the levels of 5-HT and NE, can reduce epileptic seizures and prevent the occurrence of SUDEP through multiple pathways. These pathways include increasing the seizure threshold, exerting anticonvulsant effects, and reducing the central nervous system's susceptibility to epileptiform activity 38-40. Additionally, this combination can mitigate adverse reactions caused by high effective doses of single-agent therapy. Consequently, the regulation of the 5-HT and NE systems did not simply combine their effects but created a synergistic outcome that exceeded the sum of their individual contributions. This suggested that elevating 5-HT and NE levels reduced the incidence of S-IRA through a synergistic manner.

Secondly, based on the experiments detailed above, the combined use of 5-HT and NE not only effectively reduces the incidence of S-IRA but also circumvents the adverse effects associated with high effective doses of monotherapy. Venlafaxine, a selective serotonin-norepinephrine reuptake inhibitor (SNRI), is commonly employed in the treatment of depression and has demonstrated inhibitory effects on epileptic seizures, thereby reducing their frequency. The probability of epilepsy patients developing depression tends to increase with the progression of the disease, and Venlafaxine has been shown to have a significant positive impact on alleviating depressive symptoms in these patients. Consequently, we pose the question: could venlafaxine potentially reduce the incidence of SUDEP and serve as a preventative measure? In both acoustic stimulation and PTZ models, it was observed that IP administration of venlafaxine led to a decrease in the incidence of S-IRA, reduced the duration of tonic-clonic seizures, and lowered seizure scores, all without inducing behavioral abnormalities. In our dose-response tests, venlafaxine's protective effect peaked at 50-75 mg/kg, whereas 100 mg/kg was less effective. Experimental studies in rodents have demonstrated that doses of venlafaxine at or above 75-100 mg/kg increase seizure severity and mortality following convulsant challenges 45. This proconvulsant effect is hypothesized to be mediated in part by enhanced dopaminergic receptor activation, particularly via D1 receptors, which increase cortical excitability and epileptogenic activity 45-46. In addition, high-dose venlafaxine overdose in humans has been associated with cardiac toxicity, including QT and QRS prolongation, tachycardia, and reversible cardiomyopathy, which may contribute to sudden death risk independent of seizures 47. The above might be the reasons for the increased risk of sudden death observed with high-dose venlafaxine. Notably, the inhibitory effect of venlafaxine on S-IRA was counteracted by the administration of KET or prazosin.

Furthermore, the injection of either PCPA (a serotonin synthesis inhibitor) and/or DSP-4 (a noradrenergic neurotoxin) could reverse the beneficial effects of venlafaxine on S-IRA, shorten the latency to GSz, and increase the W+C and tonic-clonic seizures. These findings underscore that venlafaxine modulates SUDEP through both the 5-HT and NE systems, with this modulation being mediated by the 5-HT2A and NE-α1 receptors. Concurrently, our observations revealed that the reduction in SUDEP incidence by venlafaxine is contingent upon the integrity of both the 5-HT and NE systems, highlighting the indispensable and concurrent role of these neurotransmitter systems in this regulatory process. We employed agonists of the 5-HT2A and α1 receptors to verify its role in the mechanism of action of venlafaxine and to elucidate the synergistic effect between the two receptors. Recent research has established the important role of TCB-2 in epilepsy and seizure-related respiratory dysfunction. In SUDEP models, TCB-2 has been demonstrated to prevent seizure-induced respiratory arrest and death through 5-HT2A receptor-mediated mechanisms 48. Additionally, TCB-2 has shown efficacy in reducing seizure-induced breathing variability following wake-occurring seizures, supporting its potential therapeutic role in preventing respiratory complications of epilepsy 49. However, research has also indicated that alpha-1 adrenergic receptor activation, the primary mechanism of phenylephrine action, promotes breathing recovery after seizure-induced respiratory arrest, which is critical for preventing seizure-induced death 50. We found that the complementary mechanisms of action exhibited by TCB-2 and phenylephrine suggested potential for synergistic therapeutic approaches in SUDEP prevention. While TCB-2 targeted the serotonergic component of respiratory control through 5-HT2A receptor activation, phenylephrine addresses the noradrenergic component through alpha-1 receptor stimulation. This dual-target approach mirrors the successful strategy demonstrated with venlafaxine but offers enhanced selectivity and potentially improved safety profiles.

Notably, IP injection of venlafaxine increased the c-fos expression in DR^5-HT^ and LC^NE^ neurons. Further calcium signal recording results showed that venlafaxine enhanced the calcium signal of DR^5-HT^ and LC^NE^ neurons. With increasing doses of venlafaxine, the activity of these neurons was suppressed, a trend consistent with behavioral changes following IP venlafaxine injection. This indicated that during the regulation of SUDEP, both DR^5-HT^ and LC^NE^ neurons were involved, showing an initial activation followed by suppression. This dynamic may be associated with the positive and negative feedback mechanisms of the drug. These results strongly suggest that both the DR^5-HT^ and LC^NE^ neurons are involved in the occurrence of SUDEP, and they appear to be interdependent, with neither being dispensable. However, the mechanisms by which the DR and LC modulate SUDEP, as well as the relationship of upstream and downstream between them, remain unclear.

Thirdly, the optogenetic and chemogenetic techniques were therefore utilized to investigate the DR^5-HT^ and LC^NE^ neurons specifically. The descending projections from the DR to the LC account for at least 50% of the 5-HT innervation of this nucleus. The LC also receives afferent neurons from the median raphe nucleus, and the DR^5-HT^ are densely innervated by LC^NE^. Inhibition of DR activity can significantly reduce the release of NE in the LC. It was found that optogenetic activation of DR^5-HT^ neurons significantly reduced the incidence of S-IRA and activated LC^NE^ neurons. Similarly, optogenetic activation of LC^NE^ neurons significantly reduced the incidence of S-IRA but did not significantly activate DR^5-HT^ neurons, a finding also confirmed by chemogenetic activation of LC^NE^ neurons. Thus, it is hypothesized that the DR^5-HT^ neurons may act as an upstream regulator of the LC^NE^ neurons. To investigate this, a forward tracing virus, HSV, was injected into the DRN, revealing that over 80% of NE neurons in the bilateral LC were labeled on average. Similarly, when a retrograde tracing virus, CTB-555, was injected into the bilateral LC, it was found that 87.52% of 5-HT neurons in the DRN were labeled on average. These findings suggest an upstream-downstream relationship between the 5-HT neurons in the DRN and the NE neurons in the LC, with the 5-HT neurons in the DRN likely also reducing the incidence of S-IRA by activating the NE neurons in the LC. Although there is evidence indicating that 5-HT neurons in the DRN of normal mice also receive dense noradrenergic innervation from the LC and are tonically activated by noradrenergic inputs, in our study, activation of NE neurons in the LC did not result in noticeable activity changes in the 5-HT neurons of the DRN. This discrepancy may be attributed to changes in neural circuit activation in the brain of SUDEP model mice during epileptic seizures. Additionally, these findings indicate that both the 5-HT neurons in the DRN and the NE neurons in the LC are capable of modulating the occurrence of SUDEP, and there exists a dependency between their actions.

Finally, how do DR^5-HT^ and LC^NE^ neurons regulate SUDEP? As our previous research on the mechanisms of DR-associated 5-HTergic and LC-associated NEergic systems in regulating SUDEP has shown, the DR and LC are pivotal relay points for the synthesis, release, and transmission of neurotransmitter signals in the monoaminergic neurotransmitter system, but they are not the final determinants in regulating SUDEP. Our extensive previous research indicated that S-IRA can induce SUDEP in acoustic stimulation or PTZ injection models of DBA/1 mouse^22,36,42^, aligning with the mainstream view that S-IRA was a primary factor in SUDEP. One critical finding of our research was the involvement of the 5-HTergic neural circuit between the DR and PBC in SUDEP. Given the pivotal role of the PBC in regulating respiratory rhythm, our focus shifted to the PBC, identifying it as the optimal candidate for the final relay point where DR-related serotonergic and LC-related noradrenergic systems synergistically regulate SUDEP. Then the neural anatomical circuit among the DR, LC, and PBC were verified by injection of anterograde tracing virus HSV into DR and the results revealed that the majority of bilateral LC^NE^ neurons and bilateral PBC neurons were labeled. Similarly, injection of retrograde tracer CTB-555 into bilateral PBC showed that most bilateral LC^NE^ neurons and DR^5-HT^ neurons were labeled. Based on previous and current research findings, DR^5-HT^ neurons, LC^NE^ neurons, and PBC neurons form a hierarchical relationship, with DR^5-HT^ neurons potentially reducing the incidence of S-IRA by simultaneously activating LC^NE^ and PBC neurons.

Previous research results have shown that activating the DR-PBC neural circuit has effectively reduced the S-IRA, prompting us to consider the significance of the LC as a relay station. Optogenetic activation of the DR-LC neural circuit and chemogenetic activation of LC^NE^ neurons both significantly reduced the incidence of S-IRA, markedly prolonged the latency, decreased the duration of tonic-clonic seizures, and lowered seizure scores, indicating that the LC was a sufficient condition for regulating the SUDEP. In exploring the necessary of LC for SUDEP regulation, DSP-4 significantly reversed the effect of optogenetic activation of the DR in reducing S-IRA, shortened the latency, and increased the duration of W+C, and the duration of tonic-clonic seizures, indicating that the effect of DR^5-HT^ neuron activation in reducing S-IRA incidence depended on the LC^NE^ neurons. More importantly, this suggested that the 5-HTergic and NEergic systems intrinsically regulated the SUDEP in a synergistic-dependent manner. To further verify the presence of a projection circuit between LC^NE^ neurons and the PBC that regulates the SUDEP, we microinjected an NE-α1R antagonist into the PBC before optogenetic activation of LC^NE^ neurons. The results specifically showed that antagonizing the PBC NE-α1 receptor reversed the effects of optogenetic activation of LC^NE^ neurons, significantly increasing the incidence of S-IRA, duration of W+C, and the duration of tonic-clonic seizures. These findings indicated that the LC was a crucial component in the DR-LC neural circuit that regulated respiratory rhythm and S-IRA via the PBC, making it both a sufficient and necessary condition for this neural circuit to regulate SUDEP. Based on previous findings, we simultaneously enhanced 5-HT and NE neurotransmission by intra-PBC injection of KET and prazosin. The results revealed that 5-HT2A and NE-α1 receptors were likely the targets through which the 5-HT and NE systems exert their synergistic-dependent effects.

## Limitations

Firstly, while we observed that a low, ineffective dose of 5-HTP combined with Atomoxetine could entirely prevent SUDEP triggered by acoustic stimulation and PTZ, suggesting that the 5-HT and NE systems are indispensable and act synergistically in modulating SUDEP, the precise mechanisms behind this interaction remain elusive and necessitate further exploration. Secondly, although our findings indicate that 5-HT neurons in the DRN and NE neurons in the LC influence SUDEP via the PBC, it is important to recognize that PBC, a critical site for regulating central respiratory rhythm, contains a diverse array of neuronal types. A multitude of neurotransmitters, such as glutamate, glycine, GABA, serotonin, and norepinephrine, are capable of adjusting the activity of respiratory neurons within the PBC. Consequently, when the DR and LC are activated, it remains uncertain whether other neural systems, apart from the noradrenergic system influencing the PBC, are concurrently activated and involved in the modulation of SUDEP.

## Conclusion

Within the monoaminergic systems implicated in SUDEP pathogenesis, our study is the first to demonstrate that serotonergic and noradrenergic systems regulate SUDEP through an intrinsic synergistic-dependent mechanism, rather than acting in isolation as previously suggested. More importantly, we identified the DR-LC-PBC circuit as a critical protective pathway, in which the PBC—a central regulator of respiratory rhythm—integrates convergent serotonergic and noradrenergic inputs via 5-HT2A and NE-α1 receptors. This receptor-specific neural communication is pivotal in suppressing S-IRA and thereby mitigating SUDEP. Clinically, the relevance of this mechanism is underscored by our pharmacological findings with venlafaxine, a widely used antidepressant that enhances 5-HT and NE signaling, which further supports the translational potential of dual monoaminergic modulation in patients with epilepsy, particularly those with comorbid depression.

These findings establish a previously unrecognized circuit- and receptor-level synergy between 5-HT and NE systems in SUDEP regulation, providing both mechanistic novelty and a promising therapeutic direction for the prevention of SUDEP.

## Supplementary Material

Supplementary tables and movie legends.

Supplementary movie 1.

Supplementary movie 2.

Supplementary movie 3.

Supplementary movie 4.

Supplementary movie 5.

Supplementary movie 6.

Supplementary movie 7.

Supplementary movie 8.

Supplementary movie 9.

Supplementary movie 10.

Supplementary movie 11.

Supplementary movie 12.

Supplementary movie 13.

Supplementary movie 14.

## Figures and Tables

**Figure 14:**
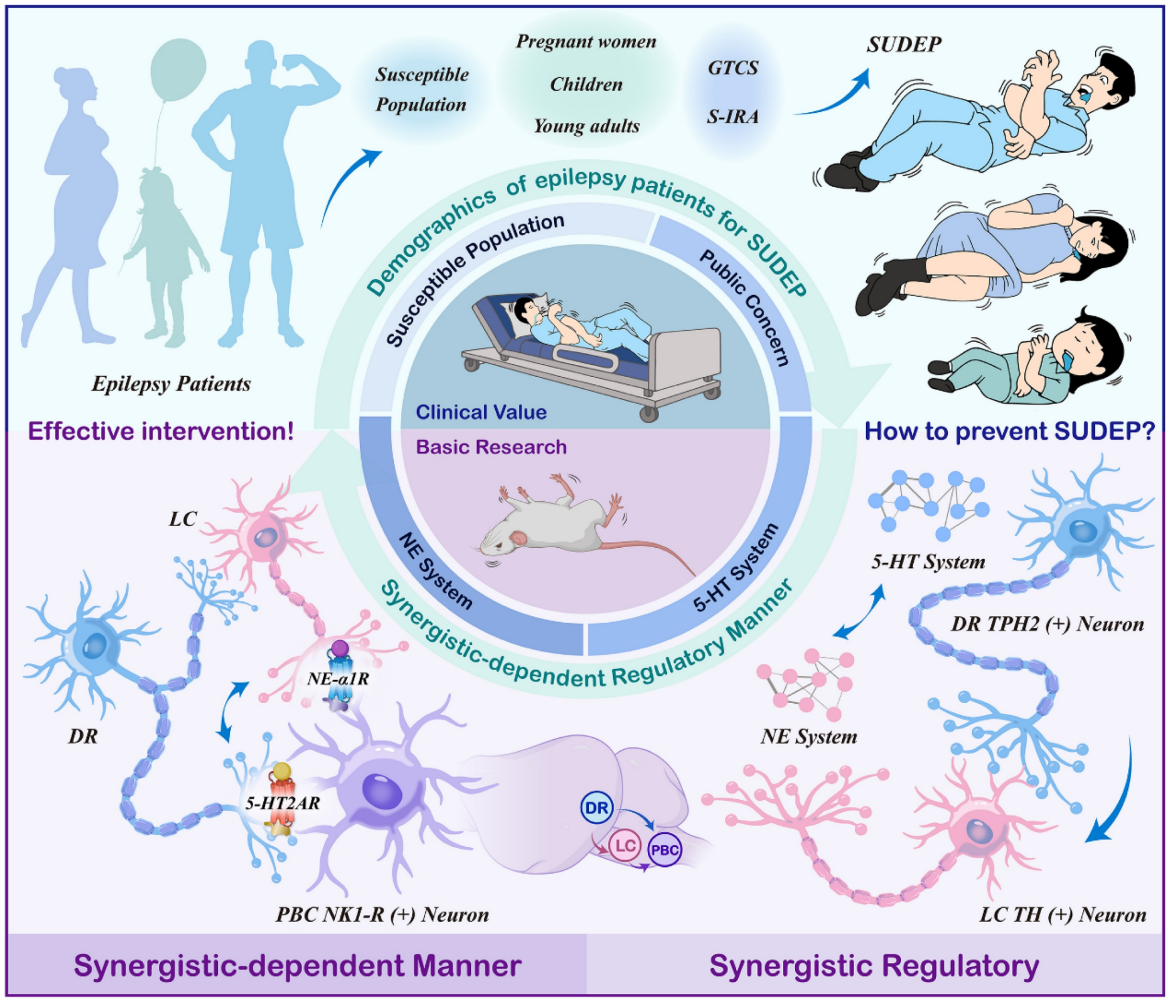
**The 5-HT and NE systems regulated SUDEP through an intrinsic synergistic-dependent manner.** From a human demographic perspective, epilepsy ranks as the second most prevalent neurological disorder, seriously affecting patients' quality of life and even increasing mortality, especially in gravidity, children and youth. As a serious public health concern, the American Epilepsy Society (AES), Partner Against Mortality in Epilepsy (PAME) and Danny Did Foundation have been established. We therefore conducted a series of studies and found that DR^5-HT^ and LC^NE^ neurons regulating SUDEP through PBC in a synergistic (left) and dependent (right) manner.
