# Supplementary material for: An efficient neural network of cooperating serotonergic and noradrenergic neurons in modulating sudden unexpected death in epilepsy
Source: Int J Biol Sci. 2025 Oct 10;21(14):6452–81. doi: 10.7150/ijbs.114659 (PMC12594605; doi:10.7150/ijbs.114659)
Supplement: Supplementary file 1 — Supplementary tables and movie legends. [file ijbsv21p6452s1.pdf]

Supplementary materials

Table S1. Summary of experimental groups of DBA/1 mice

Figure	Experimental groups	Number of mice
Figure 2	**Acoustic stimulation induced model**	
	i.p. Saline + i.p. Saline	8
	i.p. 5-HTP (100 mg/kg) + i.p. Saline	9
	i.p. 5-HTP (125 mg/kg) + i.p. Saline	9
	i.p. 5-HTP (100 mg/kg) + i.p. Atomoxetine (5mg/kg)	7
	i.p. Saline + i.p. Saline	5
	i.p. Atomoxetine (5mg/kg) + i.p. Saline	6
	i.p. Atomoxetine (10mg/kg) + i.p. Saline	5
	i.p. Atomoxetine (15mg/kg) + i.p. Saline	6
	i.p. Atomoxetine (20mg/kg) + i.p. Saline	5
	i.p. Atomoxetine (5mg/kg) + i.p. 5-HTP (100 mg/kg)	7
	**PTZ injection induced model**	
	i.p. Saline + i.p. Saline	8
	i.p. 5-HTP (100 mg/kg) + i.p. Saline	7
	i.p. 5-HTP (125 mg/kg) + i.p. Saline	8
	i.p. 5-HTP (100 mg/kg) + i.p. Atomoxetine (5mg/kg)	9
	i.p. Saline + i.p. Saline	6
	i.p. Atomoxetine (5mg/kg) + i.p. Saline	6
	i.p. Atomoxetine (15mg/kg) + i.p. Saline	7
	i.p. Atomoxetine (5mg/kg) + i.p. 5-HTP (100 mg/kg)	9
Figure 3	**Acoustic stimulation induced model**	
	i.p. Saline	6
	i.p. venlafaxine (5mg/kg)	8
	i.p. venlafaxine (15mg/kg)	6
	i.p. venlafaxine (25mg/kg)	7
	i.p. venlafaxine (50mg/kg)	7

	i.p. venlafaxine (75mg/kg)	8
	i.p. venlafaxine (100mg/kg)	7
	i.p. 25%DMSO + i.p. Saline	8
	i.p. 25%DMSO + i.p. venlafaxine	7
	i.p. KET + i.p. venlafaxine	8
	i.p. 25%DMSO + i.p. Saline	7
	i.p. 25%DMSO + i.p. venlafaxine	6
	i.p. prazosin + i.p. venlafaxine	8
	**PTZ injection induced model**	
	i.p. Saline + i.p. PTZ	7
	i.p. venlafaxine (5mg/kg) + i.p. PTZ	6
	i.p. venlafaxine (15mg/kg) + i.p. PTZ	12
	i.p. venlafaxine (25mg/kg) + i.p. PTZ	8
	i.p. venlafaxine (50mg/kg) + i.p. PTZ	8
	i.p. venlafaxine (75mg/kg) + i.p. PTZ	7
	i.p. venlafaxine (100mg/kg) + i.p. PTZ	8
	i.p. 25%DMSO + i.p. Saline	6
	i.p. 25%DMSO + i.p. venlafaxine	6
	i.p. KET + i.p. venlafaxine	6
	i.p. 25%DMSO + i.p. Saline	6
	i.p. 25%DMSO + i.p. venlafaxine	6
	i.p. prazosin + i.p. venlafaxine	6
**Figure 4**	**PTZ injection induced model**	
	i.p. Saline + i.p. Saline	7
	i.p. PCPA (1d) + i.p. Saline	6
	i.p. PCPA (5d) + i.p. Saline	5
	i.p. Saline + i.p. venlafaxine	8
	i.p. PCPA (1d) + i.p. venlafaxine	8
	i.p. PCPA (5d) + i.p. venlafaxine	6
	i.p. PCPA (5d) + i.p. DSP-4 + i.p. venlafaxine	6

	i.p. Saline + i.p. Saline	7
	i.p. DSP-4 + i.p. Saline	8
	i.p. Saline + i.p. venlafaxine	8
	i.p. DSP-4 + i.p. venlafaxine	6
	i.p. DSP-4 + i.p. PCPA (5d) + i.p. venlafaxine	6
	**DR c-fos (+)**	
	i.p. Saline	6
	i.p. venlafaxine (5mg/kg)	6
	i.p. venlafaxine (15mg/kg)	6
	i.p. venlafaxine (25mg/kg)	6
	i.p. venlafaxine (50mg/kg)	6
	i.p. venlafaxine (75mg/kg)	6
	**LC c-fos (+)**	
	i.p. Saline	7
	i.p. venlafaxine (5mg/kg)	7
	i.p. venlafaxine (15mg/kg)	7
	i.p. venlafaxine (25mg/kg)	7
	i.p. venlafaxine (50mg/kg)	7
	i.p. venlafaxine (75mg/kg)	7
	**Calcium Signal Recording: DR**	
	icv. Saline	7
	icv. venlafaxine (1.25mg/kg)	7
	icv. venlafaxine (2.5mg/kg)	7
	icv. venlafaxine (6.25mg/kg)	11
**Figure 5**	icv. venlafaxine (12.5mg/kg)	7
	icv. venlafaxine (25mg/kg)	8
	**Calcium Signal Recording: LC**	
	icv. Saline	6
	icv. venlafaxine (1.25mg/kg)	8
	icv. venlafaxine (2.5mg/kg)	9

	icv. venlafaxine (6.25mg/kg)	6
	icv. venlafaxine (12.5mg/kg)	5
	icv. venlafaxine (25mg/kg)	7
	DR no photostimulation	5
	DR photostimulation (15 mV/20 min)	7
	Bilateral LC c-fos with DR no photostimulation	5
	Left LC c-fos with DR photostimulation	5
**Figure 6**	Right LC c-fos with DR photostimulation	5
	LC no photostimulation	5
	LC photostimulation (15 mV/25 min)	6
	DR c-fos with LC no photostimulation	4
	DR c-fos with LC photostimulation	4
	i.p. Vehicle	6
**Figure 7**	i.p. CNO (0.5 mg/kg)	6
	i.p. CNO (1 mg/kg)	6
**Figure 9**	i.p. Vehicle	6
	i.p. CNO (1 mg/kg)	6
	i.p. Vehicle	5
	DR photostimulation	7
**Figure 10**	i.p. DSP-4 (1d) + DR photostimulation	8
	i.p. DSP-4 (7d) + DR photostimulation	7
**Figure 11**	i.p. Saline	6
	i.p. venlafaxine (25mg/kg)	6
	PBC 25%DMSO + DR no photostimulation	7
	PBC 25%DMSO + DR photostimulation	7
	PBC prazosin (9.528nmol) + DR photostimulation	7
**Figure 12**	i.p. Vehicle	6
	i.p. venlafaxine	7
	PBC KET (9.15nmol) + i.p. venlafaxine	6
	PBC prazosin (3.19nmol) + i.p. venlafaxine	5

	PBC prazosin + PBC KET + i.p. venlafaxine	5
Figure 13	i.p. Vehicle	6
	i.p. TCB-2 (2.5mg/kg)	6
	i.p. TCB-2 (5mg/kg)	6
	i.p. Phenylephrine (6mg/kg)	6
	i.p. Phenylephrine (12mg/kg)	6
	i.p. Phenylephrine (24mg/kg)	6
	i.p. Phenylephrine (48mg/kg)	6
	PBC TCB-2 (35.4mmol)	6
	PBC TCB-2 (70.8mmol)	6
	PBC Phenylephrine (0.7mol)	6
	PBC Phenylephrine (1.4mol)	6
	C57	6
	DBA/1	6

Table S2. Statistical analysis in each group

Figure	Comparisons	Test
**Figure 2**	Incidence of S-IRA	Wilcoxon signed-rank test
	AGSz/GSz latency	Ordinary one-way ANOVA
	Duration of W+C	Ordinary one-way ANOVA
	Duration of Tonic-clonic	Ordinary one-way ANOVA
	Seizure Scores	Ordinary one-way ANOVA
**Figure 3**	Incidence of S-IRA	Wilcoxon signed-rank test
	AGSz/GSz latency	Ordinary one-way ANOVA
	Duration of W+C	Ordinary one-way ANOVA
	Duration of Tonic-clonic	Ordinary one-way ANOVA
	Seizure Scores	Ordinary one-way ANOVA
**Figure 4**	Incidence of S-IRA	Wilcoxon signed-rank test
	GSz latency	Ordinary one-way ANOVA
	Duration of W+C	Ordinary one-way ANOVA
	Duration of Tonic-clonic	Ordinary one-way ANOVA
	Seizure Scores	Ordinary one-way ANOVA
	Quantification of c-fos (+)/TPH2 (+) section	Unpaired t-test
	Quantification of c-fos (+)/TH (+) section	Unpaired t-test
**Figure 5**	Incidence of S-IRA	Wilcoxon signed-rank test
	ΔF/F peak	Unpaired t-test
**Figure 6**	Incidence of S-IRA	Wilcoxon signed-rank test
	GSz latency	Ordinary one-way ANOVA
	Duration of W+C	Ordinary one-way ANOVA
	Duration of Tonic-clonic	Ordinary one-way ANOVA
	Seizure Scores	Ordinary one-way ANOVA
	Quantification of c-fos (+)/TPH2 (+) section	Unpaired t-test
	Quantification of c-fos (+)/TH (+) section	Unpaired t-test
**Figure 7**	Incidence of S-IRA	Wilcoxon signed-rank test
	GSz latency	Ordinary one-way ANOVA

	Duration of W+C	Ordinary one-way ANOVA
	Duration of Tonic-clonic	Ordinary one-way ANOVA
	Seizure Scores	Ordinary one-way ANOVA
	Quantification of c-fos (+)/TH (+) section	Unpaired t-test
**Figure 9**	Incidence of S-IRA	Wilcoxon signed-rank test
	ΔF/F peak	Unpaired t-test
**Figure 10**	Incidence of S-IRA	Wilcoxon signed-rank test
	GSz latency	Ordinary one-way ANOVA
	Duration of W+C	Ordinary one-way ANOVA
	Duration of Tonic-clonic	Ordinary one-way ANOVA
	Seizure Scores	Ordinary one-way ANOVA
**Figure 11**	Quantification of c-fos (+)/TH (+) section	Unpaired t-test
	Quantification of c-fos (+)/TPH2 (+) section	Unpaired t-test
**Figure 12**	Incidence of S-IRA	Wilcoxon signed-rank test
	GSz latency	Ordinary one-way ANOVA
	Duration of W+C	Ordinary one-way ANOVA
	Duration of Tonic-clonic	Ordinary one-way ANOVA
	Seizure Scores	Ordinary one-way ANOVA
**Figure 13**	Incidence of S-IRA	Wilcoxon signed-rank test
	GSz latency	Ordinary one-way ANOVA
	Duration of W+C	Ordinary one-way ANOVA
	Duration of Tonic-clonic	Ordinary one-way ANOVA
	Seizure Scores	Ordinary one-way ANOVA

Table S3. Reagent or resource

Reagent or resource	Source	Identifier
**Antibodies**		
Mouse anti-TH	Merck-Millipore	MAB318
Mouse anti-TPH2	Sigma-Aldrich	T0678
Rabbit anti-c-fos	Cell Signaling Technology	2250S
Donkey anti-mouse Alexa 546	Thermo Fisher Scientific	A10036
Donkey anti-mouse Alexa 488	Thermo Fisher Scientific	A21202
Goat anti-rabbit Cy5	Thermo Fisher Scientific	A10523
**Bacterial and virus strains**		
AAV2/9-mCaMKIIa-GCaMP6f-WPRE-pA	Brain VTA Technology	N/A
rAAV-DBH-GCaMP6m-WPRE-hGH pA	Brain VTA Technology	N/A
pAAV-TPH2 PRO-ChETA-EYFP-WPRES-PAS	Shengbo	N/A
pAAV-CAG-DIO-CHETA-EGFP	Shengbo	N/A
mTH-Cre-AAV+ AAV-EF1a-DIO-hM3Dq-mCherry	Brain VTA Technology	N/A
HSV-1 H129	Brain VTA Technology	N/A
mTH-Cre-AAV+ AAV-EF1a-DIO-hChR2 (H134R)-eYFP	Brain VTA Technology	N/A
**Chemicals, peptides, and recombinant proteins**		
PTZ	Sigma-Aldrich	Cat #P6500
5-HTP	Sigma-Aldrich	Cat #107751
Atomoxetine	Sigma-Aldrich	Ca #Y0001586
venlafaxine	Sigma-Aldrich	*P*HR1736
DSP-4	Sigma-Aldrich	C8417
PCPA	Sigma-Aldrich	C3635
KET	Sigma-Aldrich	Cat #8006
prazosin	Sigma-Aldrich	Cat # P7791

DMSO	Sigma-Aldrich	SHBK2703
Clozapine N-oxide (CNO)	Brain VTA Technology Co., Ltd.	N/A
CTB-555	Brain VTA Technology Co., Ltd.	N/A
**Experimental models: Organisms/strains**		
TH-Cre C57BL/6J mice	Jackson Lab	B6.Cg-7630403G23RikTg (TH-Cre) 1Tmd/J
**Software and algorithms**		
Fiber photometry system	Inper	C11946
ImageJ	NIH, Bethesda, MD, USA	https://imagej.nih.gov/ij/
GraphPad Prism TM8.0		
SPSS version 23.0		

Supplemental movies

Movies	Content	Related Figure
**Pharmacology experiments**		
**Movie 1**	An example of DBA/1 mice intervened with acoustic stimulation, and saline, 100mg/kg 5-HTP, 5mg/kg Atomoxetine 100mg/kg or 5-HTP plus 5mg/kg Atomoxetine, observing behavioral changes.	**Figure 2**
**Movie 2**	An example of DBA/1 mice intervened with PTZ, and saline, 100mg/kg 5-HTP, 5mg/kg Atomoxetine 100mg/kg or 5-HTP plus 5mg/kg Atomoxetine, observing behavioral changes.	
**Movie 3**	An example of DBA/1 mice intervened with acoustic stimulation, and saline or 25 mg/kg venlafaxine, observing behavioral changes.	**Figure 3**
**Movie 4**	An example of DBA/1 mice intervened with PTZ, and saline or 25 mg/kg venlafaxine, observing behavioral changes.	
**Movie 5**	An example of DBA/1 mice intervened with 25mg/kg venlafaxine and PTZ, and with or without 20 mg/kg KET, observing behavioral changes.	
**Movie 6**	An example of DBA/1 mice intervened with 25mg/kg venlafaxine and acoustic stimulation, and with or without 20 mg/kg KET, observing behavioral changes.	
**Movie 7**	An example of DBA/1 mice intervened with 25 mg/kg venlafaxine and acoustic stimulation, and with or without 0.01 mg/kg prazosin, observing behavioral changes.	
**Movie 8**	An example of DBA/1 mice intervened with PTZ, and with or without 0.01 mg/kg prazosin and 25 mg/kg venlafaxine, observing behavioral changes.	

**Movie 9**	An example of DBA/1 mice intervened with PTZ, and with or without 800 mg/kg PCPA (1 day) or 25 mg/kg venlafaxine, observing behavioral changes.	**Figure 4**
**Movie 10**	An example of DBA/1 mice intervened with PTZ, and with or without DSP-4 or 25 mg/kg venlafaxine, observing behavioral changes.	
**Optogenetic experiments**		
**Movie 11**	An example of TH-Cre DBA/1 mice intervened with PTZ, and with or without photostimulation to DR, observing behavioral changes.	**Figure 6**
**Movie 12**	An example of TH-Cre DBA/1 mice intervened with PTZ, and with or without photostimulation to LC, observing behavioral changes.	
**Movie 13**	An example of TH-Cre DBA/1 mice intervened with PTZ, DSP-4 and photostimulation to DR, observing behavioral changes.	**Figure 10**
**Chemogenetic experiments**		
**Movie 14**	An example of DBA/1 mice intervened with PTZ, and with or without the chemogenetic activation of the LC^{NE} neurons, recording the calcium signaling of PBC.	**Figure 9**
